# Quantification and localization of integrated HIV-1 in memory and naïve CD4+ T cells from adolescents and young adults with perinatally-acquired HIV-1

**DOI:** 10.1371/journal.ppat.1014369

**Published:** 2026-07-13

**Authors:** Adit Dhummakupt, Alexander G. McFarland, Joseph Szewczyk, John Everett, Carole Lee, Aoife M. Roche, Soumia Bekka, Thuy Anderson, Allison L. Agwu, Frederic D. Bushman, Deborah Persaud

**Affiliations:** 1 Johns Hopkins University School of Medicine, Department of Pediatrics, Division of Infectious Diseases, Baltimore, Maryland, United States of America; 2 University of Pennsylvania Perelman School of Medicine, Department of Microbiology, Philadelphia, Pennsylvania, United States of America; National Cancer Institute, UNITED STATES OF AMERICA

## Abstract

HIV-1 establishes latency in resting memory CD4+ T cells, creating a long-lived reservoir that poses a barrier to HIV-1 cure. This reservoir is thought to form by initial infection of antigen-activated CD4+ T-cells that then transition into quiescent memory CD4+ T cells. However, naïve CD4+ T cells have been shown to harbor integrated HIV-1 in adults with non-perinatally acquired HIV-1, and more recently in young children. Given that perinatal HIV-1 transmission is established in an immune environment dominated by naïve CD4+ T cells, we hypothesized that naïve CD4+ T cells may be a substantial reservoir in young adults with longstanding perinatal infection. To investigate this, we studied a cohort of 10 adolescents and young adults (AYA) living with well-controlled, perinatally-acquired HIV-1. We quantified and characterized intact and defective HIV-1 proviruses and their location in sorted naïve and memory CD4+ T cells using an intact proviral DNA assay, near full-length single genome sequencing, and integration site profiling. We found that a median of 93.5% of HIV-1 proviruses resided in memory CD4+ T cells in AYA with perinatal HIV-1, with a few participants also having intact proviruses in naïve CD4+ T cells, but at substantially lower frequencies. HIV-1 integration site analysis, using the unbiased ligation-mediated PCR method, showed that 78.0% of proviruses were located within active transcription units in introns and 46.9% of integration sites were clonal, with detection in multiple cells. Overall, similar to adults with non-perinatally acquired HIV-1, integration sites were enriched in regions with epigenetic marks for active enhancers and transcription. Therapies to attain HIV-1 ART-free remission and cure for this population will need to consider reservoirs in non-canonical T cells subsets, as well as substantial HIV-1 infected cells arising from cellular proliferation.

## Author summary

Adolescents and young adults (AYA) living with perinatally acquired HIV-1 are a unique cohort, having lived for decades with HIV-1. The establishment of a latent reservoir in infancy, when the immune environment is comprised of abundant naïve CD4+ T cells, distinguishes perinatal transmission of HIV-1. Thus, the cellular location and sites of integration may differ with longstanding perinatally acquired HIV-1 compared with adulthood. In this study, we examined the relative abundance of HIV-1 in naïve and memory CD4+ T cells through quantification of inferred intact and defective proviruses in the naïve and memory CD4+ T cells and their sites of HIV-1 integration. In these AYA living with well suppressed perinatal HIV-1, the majority of proviral genomes resided within memory CD4+ T cells, with evidence of clonal expansion and persistence largely within active genes in introns. Naïve CD4+ T cells also had HIV-1 proviruses with evidence of clonal expansion. Thus, the reservoir for HIV-1 in longstanding treated perinatal infection is mainly within memory CD4+ T cells, with a lesser contribution of naïve CD4+ T cells, demonstrating similar features of HIV-1 persistence in AYA with perinatal HIV-1 as in adults with non-perinatally-acquired HIV-1.

## Introduction

Inducible, replication-competent HIV-1 proviruses persist primarily in resting memory CD4+ T cells in adults, forming a latent reservoir that prevents viral eradication [1–3]. The reservoir forms largely through antigen-driven immune activation of CD4+ T cells, followed by the development of immune memory [4]. During the process of transitioning to resting memory CD4+ T cells, HIV-1 proviruses become transcriptionally silent, evading immune detection, but remain inducible and capable of driving rebound viremia in the absence of antiretroviral therapy (ART) [5]. The stability of the reservoir is due to the long-lived nature of memory CD4+ T cells and their capacity to undergo homeostatic proliferation and clonal expansion [6–8], providing a major barrier to ART-free remission and cure, for which target therapies are under investigation [9].

Recent approaches to studying HIV-1 persistence under ART to inform efficacy of ART-free remission and cure interventions include the Intact Proviral HIV-1 DNA Assay (IPDA) [10–13], which quantifies double-positive, inferred intact proviruses and single-positive, defective proviruses within the total pool of infected cells. Proviral structure and diversity during ART can be further defined using near full-length single genome sequencing (nFLSGS) [14,15], and integration site profiling [16].

The capacity for HIV-1 proviruses to be induced to produce virions, along with evidence of clonal amplification from CD4+ T cell proliferation, can be inferred from the location and orientation of HIV-1 integration in the human genome. In adults, these analyses demonstrated that memory CD4+ T cells are the principal reservoir for HIV-1 [17,18]. More recent studies, however, identified a smaller reservoir for HIV-1 in naïve CD4 + T cells in adults [19–22], indicating that HIV-1 latency can be established through mechanisms beyond antigen-induced immune activation. In infants and children, naïve CD4 + T cells comprise >90% of the CD4 + T cell pool, making them a greater potential to contribute to the latent reservoir pool [23]. Consistent with this is that naïve CD4 + T cells were shown to harbor the majority of both SIV and SHIV DNA in the infant non-human primate (NHP) models [24,25], supporting direct infection of naïve CD4 + T cells and a potential reservoir in perinatal infection. However, with age, naïve CD4 + T cells frequencies decline as antigen exposure drives the formation of the memory CD4 + T cell compartment [26]. Thus, memory CD4 + T cells become the predominant population in childhood and adolescence [27]. It has recently been shown that even in young children and adolescents, the majority of proviruses are indeed in memory CD4 + T cells [28,29], suggesting that the perinatal HIV-1 reservoir is dynamic in childhood. It is therefore critical to quantify the distribution of naïve and memory CD4 + T cells to the reservoir in perinatal HIV-1 across the ages to guide therapeutic strategies aimed at eliminating HIV-1 reservoirs for ART-free remission and cure in this growing population.

We examined the distribution of HIV-1 proviruses across naïve and memory CD4 + T cell subsets from adolescents and young adults (AYA) with perinatal HIV-1, who have maintained long-term virologic suppression since childhood. Furthermore, using the Intact Proviral DNA Assay (IPDA), we quantified inferred intact and defective proviruses and defined their structure and integration landscape.

## Results

We studied ten AYA with perinatal HIV-1 enrolled in an ongoing cohort study evaluating HIV-1 persistence during long-term suppressive ART. Table 1 summarizes the demographic, immunologic, and virologic features of the cohort. The median age at study was 15.4 years (Interquartile Range (IQR) 14.2 – 17.7), with a median duration of virologic suppression (DVS) of 7.8 years (IQR 6 – 14.6). Eighty percent (80%) of the participants were female; 80% Black, 10% White/Mixed, and 10% Asian. Seven participants (70%) had subtype B HIV-1; the remaining participants had subtypes C, A/E and A/G.

**Table 1:** Participant characteristics.

Partici-pant ID	Age at Analysis (Years)	Sex	Race	HIV-1 Subtype	CD4 + count (cells/mm^3^) near time of analysis^~^	CD4 + Nadir (cells/mm^3)^	Antiretroviral Therapy at analysis	Duration of Virologic Suppress-ion (Years)	Age at Virologic Suppression
0113*	15.7	Male	Black	B	439	213	TAF/FTC/ EVG/C	2.9	12.8
0117	17.7	Female	Black	C	884	196^~^	ABC/3TC/DTG	8.2	9.5
0300	18.2	Female	Black	B	869	384	TAF/FTC/BIC	16.1	2.1
0301	23.6	Female	Black	B	758	300	TAF/FTC/BIC	7.4	16.2
0304	17.4	Female	Black	A/G	774	533^~^	ABC/3TC/DTG	14.6	2.8
0305	14.2	Female	Asian	A/E	981	227	TAF/FTC/RPV	8.4	5.8
0306	11.5	Female	Black	B	696	374	TAF/FTC/BIC	4.1	7.4
0307	10.0	Female	White/Mixed	B	1784	1315	TAF/FTC/BIC	4.1	5.9
M0105	15.0	Female	Black	B	785	740^~^	ABC/3TC/DTG	6.0	9.0
M0113	15.0	Male	Black	B	608	715^~^	ABC/3TC/DTG	15.0	0.0

~ CD4 + T cell count was performed within a week of study sample collection;

*Suppressed for 12.4 years with brief ART interruption for 4 weeks before re-suppression for 2.85 years before study; TAF: tenofovir alafenamide, FTC: emtricitabine, EVG: Elvitegravir, C: cobicistat, ABC: abacavir, 3TC: lamivudine, DTG: dolutegravir, BIC: bictegravir, RPV: Rilpivirine;

Naive CD4 + T cells (CCR7 + CD45RA + CD28 + CD95-) comprised a median of 48.3% (IQR 37.9 – 68.0) of total CD4 + T cells, with the highest proportion (86.4%) observed in the youngest participant (age 10 years; S1 Fig). Among memory subsets, central memory CD4 + T cells accounted for a median of 28.9% (IQR 16.4 – 46.4), transitional memory for 7.2% (IQR 5.3 – 11.8), and effector memory for 0.7% (IQR 0.3 – 0.9) of total CD4 + T cells. The median numbers of naïve and memory CD4 + T cells obtained after cell sorting from a median of 10 x 10^6^ (IQR 8.0 – 12 x 10^6^) purified CD4 + T cells were 3.25 x 10^6^ (IQR 1.4 – 2.6 x 10^6^) and 2.11 x 10^6^ (IQR 2.3 – 3.9 x 10^6^), respectively.

**Fig 1:**
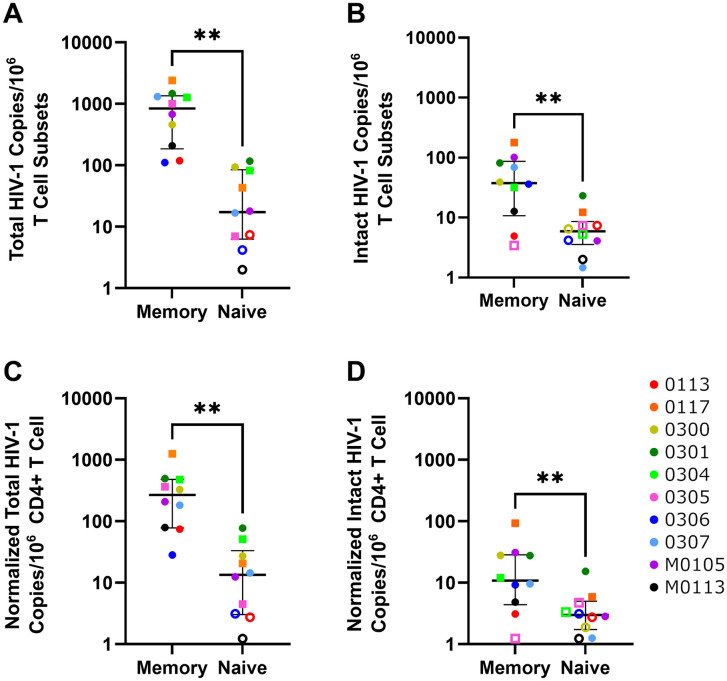
Copies of total and intact copies per million cells by a multiplexed ddPCR assay. **A)** Total HIV-1 copies per 10^6^ cells. **B)** Inferred Intact HIV-1 copies per 10^6^ cells. Copies per 10^6^ cells were divided by the percentage of respective cell populations to give **C)** total and **D)** inferred intact HIV-1 copies per 10^6^ CD4 + T cells in the memory and naïve populations, normalized by percent memory and intact CD4 + T cells. Circles: subtype B; squares: non-subtype **B.** Open shapes: undetectable, value represents LOD. Significance was assessed with paired Wilcoxon test. **: p < 0.005.

HIV-1 DNA was detected in memory CD4+ T cells in all 10 participants quantified from a median of 2.6 x 10^5^ cells (IQR 1.4 – 2.9 x 10^5^); the median total HIV-1 DNA was 840.9 copies/10^6^ cells (IQR 185.7-1356.0, Fig 1A). HIV-1 DNA was also detected in naïve CD4+ T cells of seven participants, assessed from a median of 2.3 x 10^5^ (IQR 1.1 – 3.6 x 10^5^; Fig 1A) cells, with a significantly lower median frequency at 17.3 copies/10^6^ cells (IQR 6.3 – 85.0 copies/10^6^ cells; p = 0.002).

We next quantified inferred intact proviruses (double-positive gag and env) using the Intact Proviral DNA Assay [6]. For the three participants with non-subtype B HIV-1, inferred intact proviruses were also defined as positive amplification of psi and env, which predicts high likelihood of intactness even in non-subtype B infections [13,30,31]. Inferred intact HIV-1 DNA was detected in the 9 of 10 participants, with a median of 37.6 copies/10^6^ memory CD4 + T cells (IQR 10.8 – 86.6, Fig 1B). Inferred intact proviruses were also detected from four of the seven participants with detectable HIV-1 DNA in the naïve CD4 + T cells (0117, 0301, 0307, M0105), at a median of 5.9 copies/10^6^ cells (IQR 3.6 – 8.6), significantly lower than levels observed in memory CD4 + T cells (p = 0.03, Fig 1B).

Given the wide variation in naïve and memory CD4 + T cell proportions in the participants, the reservoir in naïve CD4 + T cells could be underestimated without normalizing proviral frequencies to each individual’s CD4 + T cell subset distribution. By accounting for the percent naïve and memory CD4 + T cells, inferred intact and total HIV-1 DNA can be quantified for their contributions to the total infected CD4 + T cell pool (Fig 1C-1D). After normalization, using each participant’s naïve and memory CD4 + T cell percentages, memory CD4 + T cells remained the main location for HIV-1 proviruses, with a median of 267.3 copies/10^6^ memory CD4 + T cells (IQR 78.0 – 480.8), compared with 13.4 copies/10^6^ naïve CD4 + T cells (IQR 3.0 – 33.3, p = 0.002; Fig 1C).

Normalization shows that a median of 93.5% (IQR 90.2 – 98.4) of total HIV-1 DNA resided in memory CD4 + T cells. Inferred intact proviral load remained higher in memory CD4 + T cells as well (median 10.8 copies/10^6^ cells, IQR 4.4 – 28.6) compared with naïve CD4 + T cells (median 3.0 copies/10^6^ cells, IQR 1.7 – 5.0; p = 0.03; Fig 1D).

As the proviral HIV-1 levels in naïve CD4 + T cells were low compared to the memory CD4 + T cell values, a conservative gating strategy was employed to minimize memory CD4 + T cell contamination. In a representative participant (Participant 0117), approximately 2% of cells sorted as naïve were identified as memory CD4 + T cells, primarily CD45 + TEMRAs (S2 Fig). Using an estimated 2% contamination rate, the 95% confidence interval of HIV-1 in the naïve population was corrected for memory cell contamination. Among the seven participants with detectable HIV-1 DNA in naïve CD4 + T cells, four participants reached levels that were non-detected or near the limit of detection. Nevertheless, three participants (0300, 0301, and 0304) retained high corrected levels (81.4, 82.6, and 52.1 copies/10^6^ naive cells), supporting persistence of HIV-1 in naïve CD4 + T cells with perinatal HIV-1 (S1 Table). For participants 301 and 117, this also corresponded to a detectable inferred intact proviral reservoir in naïve CD4 + T cells (5.3 and 20.4 copies/10^6^ naïve cells, respectively; S2 Table), consistent with the persistence of intact proviruses in naïve CD4 + T cells during long-term ART in a subset of AYA with perinatal HIV-1.

**Fig 2:**
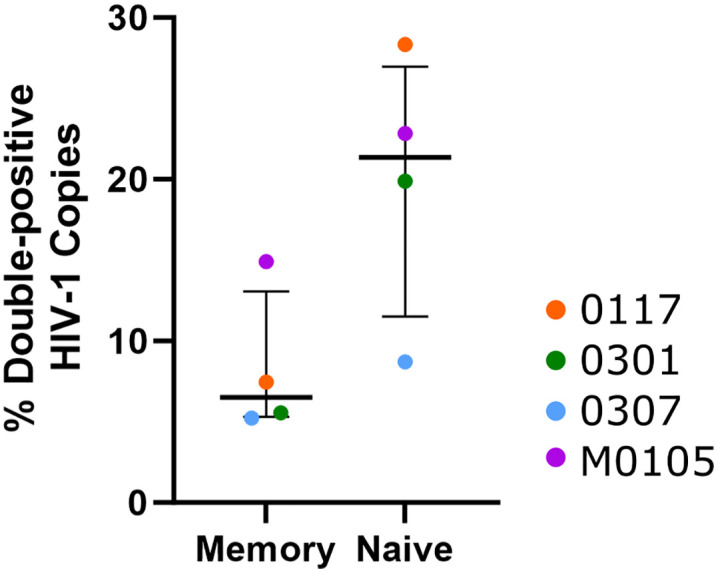
Percentage of inferred intact provirus in either memory or naïve CD4+ T cells. Participants with undetectable intact proviruses in either cell populations were eliminated for this analysis.

Of the participants with quantifiable inferred intact proviruses in both memory and naïve CD4 + T cells, the proportion of double-positive HIV-1 DNA as a function of the total HIV-1 DNA was higher in naïve CD4 + T cells (median 21.3% intact, IQR 11.5 – 27.0) than in the memory CD4 + T cells (median 6.5% intact, IQR 5.3 – 13.1, p = 0.125; Fig 2), suggesting differential maintenance of intact HIV-1 DNA in the naïve CD4 + T cell population that cannot be accounted for with memory cell contamination.

**Fig 3:**
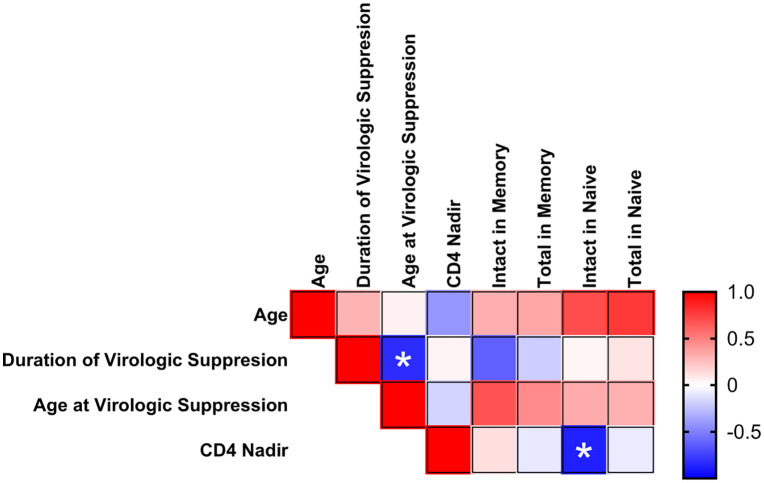
Correlations of HIV-1 inferred intact and total HIV-1 DNA by participant demographics, age and duration of virologic suppression, and CD4 nadir. AVS: age at virologic suppression, DVS: duration of virologic suppression. Significant correlations was assessed with Spearman Rank. *: p < 0.05.

Proviral loads in both CD4 + T cell subsets were correlated to participant demographics (Fig 3). Inferred intact HIV-1 DNA in naïve CD4 + T cells was inversely correlated to CD4 nadir (p = 0.012). The inferred intact proviral load in memory CD4 + T cells subsets trended towards a positive correlation with age at virologic suppression, and a negative correlation with duration of virologic suppression. Activation and exhaustion in T cell subsets were previously described in this cohort, although the flow cytometry was performed on different visits (S3 Fig) [32]. There were no significant correlations after FDR adjustment; however, the strongest correlations to proviral load across the T cell subsets were positively correlated with the proportions of CD25 + cells and inversely with proportions of CD69 + cells.

**Fig 4:**
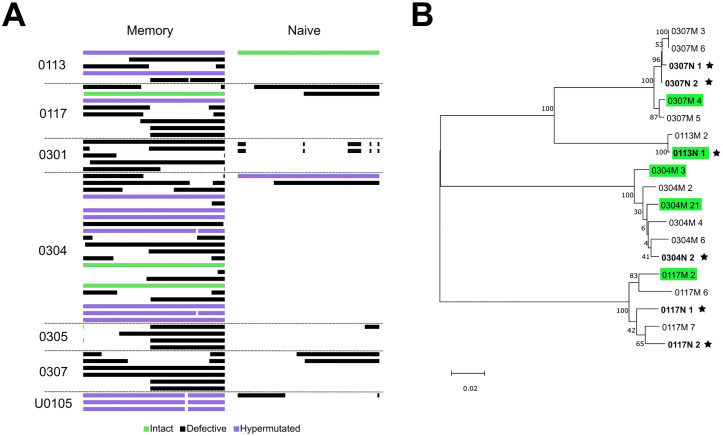
HIV-1 Proviral landscape in memory and naïve CD4+ T cells. **A)** Alignment of HIV-1 sequences using limiting dilution nFLS from either memory or naïve CD4 + T cells. Inference of intactness was performed by HIVseqinR, and manually confirmed. **B)** Maximum likelihood phylogeny tree of non-hypermutated env sequences, with 500 bootstraps. Sequences highlighted in green are designated intact by HIVseqinR, bolded sequences with stars are sequences obtained from naïve CD4 + T cells.

Sufficient genomic DNA for near-full length single genome sequencing was available for analyses in seven participants, allowing for more direct analyses of the intact proviruses. Across these individuals, 64 proviral sequences were recovered (53 from memory and 11 from naïve CD4 + T cells (Fig 4A). Most sequences (92%) were defective predominantly due to large deletions (70%) or hypermutations (22%) in both subsets. Intact proviruses were detected in memory CD4 + T cells from three of seven participants (43%), and in naïve cells from one of four participants (25%), although the contributions of potential memory T cell contamination in the naïve CD4 + T cell fraction cannot be excluded.

Among the four participants with NFLSGS in both subsets, phylogenetic analyses of env demonstrated co-mingling of the sequences from naïve and memory CD4 + T cells (Fig 4B). Tropism prediction of intact env sequences (n = 19; 13 memory and 6 naive) were predicted to be R5-tropic as determined using WebPSSM [33,34].

We analyzed HIV-1 integration sites in total CD4 + T cells from eight participants, memory CD4 + T cells from four participants and naïve CD4 + T cells from three participants using ligation-mediated PCR [35,36]. In total, 748 integration sites were identified; 384 in total CD4 + T cells, 358 in memory cells, and 6 in naïve cells, representing 474 unique sites (S3 Table).

**Fig 5:**
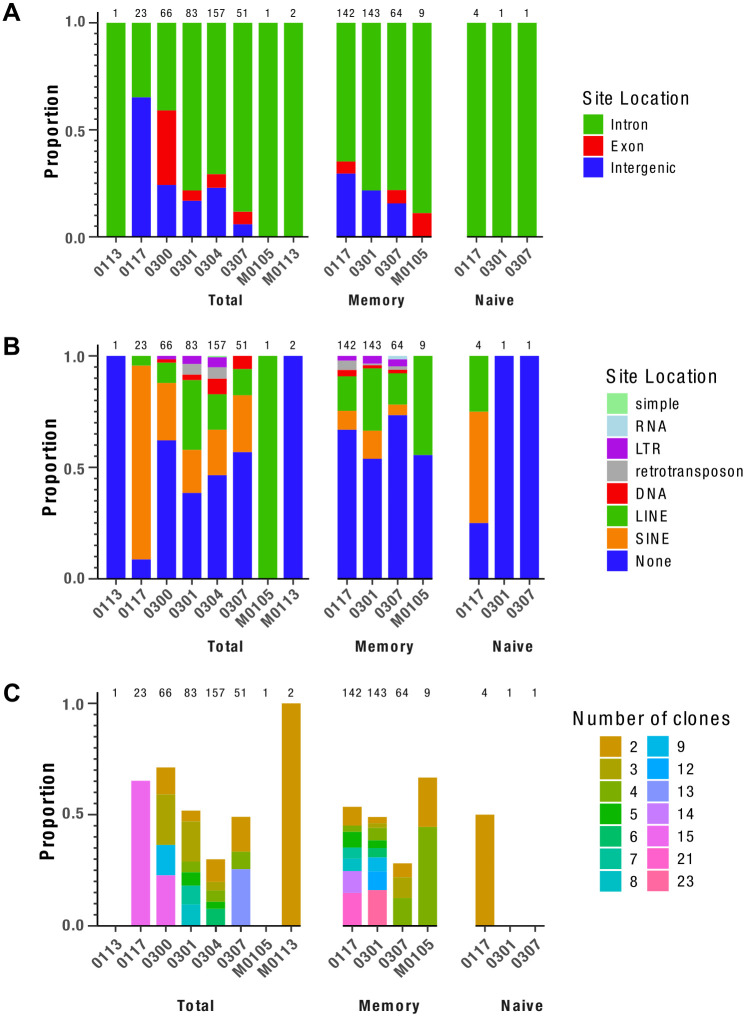
Integration site analysis in total, memory and naïve CD4+ T cells. **A)** Ratio of location of total integration sites, separated by cell populations. Number above bars represent total integration sites. **B)** Ratio of integration sites in repeat elements. **C)** Number of integration sites detected, separated by frequency of clones.

Most integrations (78%) were within transcription units, consistent with HIV-1 preference for integration into active genes [16], while 22% were located in intergenic regions (Fig 5A). Among integrations within transcription units, 91.2% mapped to introns, as expected given their predominance in the gene structure. Of integration sites located within transcription units, 48% were oriented antisense to the host gene. Many integration sites mapped to repeat regions, including SINEs, LINEs, and hAT DNA transposons (Fig 5B).

Several integration sites were detected more than once; nearly half (46.9%) were detected in multiple cells, indicating clonal proliferation (Fig 5C). Among the 581 integration events located within genes, 334 unique genes were identified (S4 Fig). Several Integrations occurred in genes associated with HIV-1 persistence. Of the three genes with integration sites across three participants (BACH2, RXFP2 and STAT5B in participants 0117, 0301 and 0307, S3 Table), STAT5B and BACH2 have been linked to clonal survival and proliferation [37]. Integration into BACH2 was detected in seven unique integration sites, including one in participant 0307 that was found in 3 clones. Of note, the largest clone detected in a single participant (participant 0301) was in VAV1 (S4 Fig), which has previously been shown to be significantly enriched in CAR T cell immunotherapy and has been associated with promoter insertional mutagenesis [38]. Integration into genomic regions associated with transcriptional silencing, including heterochromatic ZNF genes and alphoid repeats [39,40] were also detected in both total and memory CD4+ T cells from five participants, including 15 clonal sites near ZNF785, the third highest clonal population detected. In the naïve subset, one participant (0117) had a detectable clone among the three integration sites identified. When the percentage of detected singletons was compared to age at and duration of virologic suppression, we found a trend of negative correlation between duration of virologic suppression and percentage of singletons (p = 0.062). As a result, the number of clone sites detected increased with duration of virologic suppression (S5 Fig).

**Fig 6:**
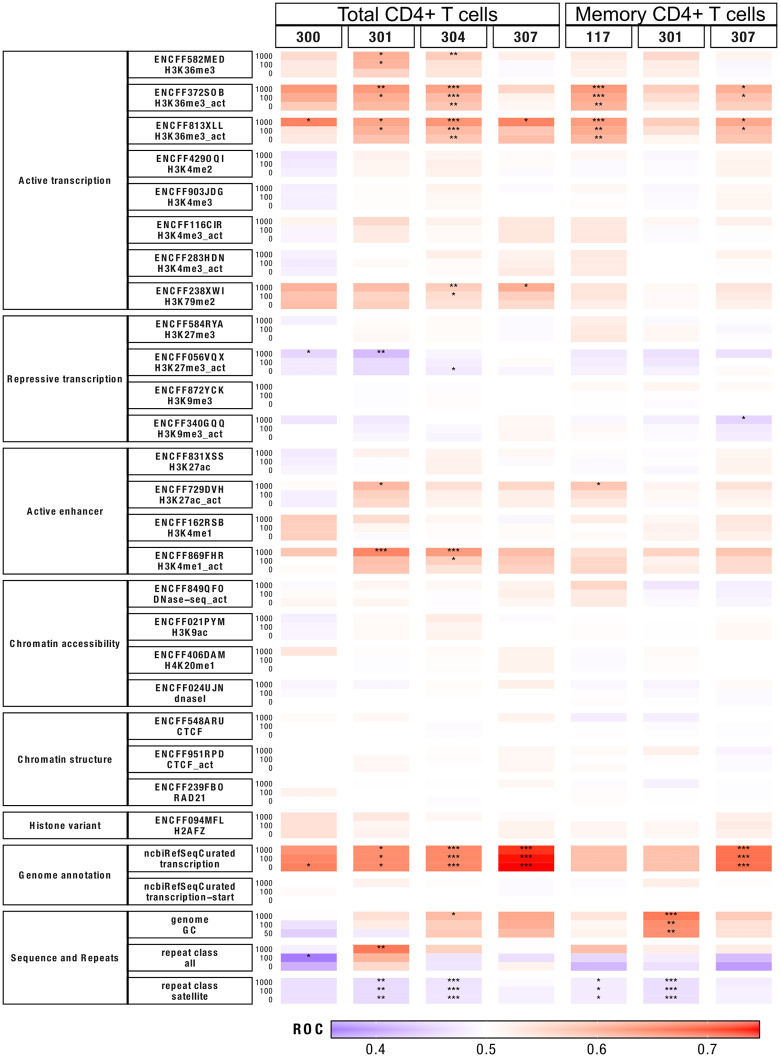
Integration site enrichment genomic and epigenetic features obtained from publicly available datasets. For each sample, the number of integration sites within each feature and window size are compared to a random distribution to generate an ROC. An ROC greater than 0.5 indicates a sample’s integration sites occur within the given feature and window size at a higher rate compared to random while an ROC less than 0.5 indicates a rate lower than random. Window sizes show degree of expansion of track boundaries. A window size of zero uses unchanged track bounds while increasing sizes expand the start and ends of tracks by half the displayed amount. Significant Benjamini-Hochberg-corrected Chi-square p-values are displayed with asterisks (p-value < 0.05 *, 0.01 **, 0.001 ***). ROC: receiver operator characteristic. Act: dataset derived from activated T cells.

Integration sites in the memory and total CD4+ T cells in participants with sufficient integration sites were correlated to publicly available epigenetic databases (Fig 6). The integration sites were generally enriched in regions with activating and enhancing marks such as acetylation, H3K36me3, H3K4me1 and HeK79me2. Conversely, integration sites were inversely correlated to heterochromatic marks, such as H3K27me3, H3K9me3 and satellite regions. These correlations were especially pronounced in activated T cells. When the epigenetic markers at integration sites were compared to an adult cohort, both cohorts were preferentially integrated into regions with marks for active transcription and were reduced in regions with repressive transcription (S6 Fig). However, H3K36me3, the most abundant activating mark associated with integration sites, was on average higher in adults than in AYA with perinatal HIV-1, although this was not significant. Finally, integration sites were enriched for specific cellular pathways (S7 Fig). Gene Ontology analysis revealed significant enrichment in pathways related to coregulator activity, nuclear receptor binding, and histone-associated functions. These patterns may reflect preferential integration into actively transcribed genes, or selection for cells with specific phenotypes arising from insertional mutagenesis.

## Discussion

In this study of 10 AYA ages 10.0 to 23.6 with longstanding perinatal HIV-1 who maintained virologic suppression from 6 – 16 years of life with ART, we detected that most HIV-1 proviruses reside in memory CD4 + T cells, making this the predominant T cell subset to maintain the reservoir in this group. However, both inferred intact and defective HIV-1 DNA was detected in naïve CD4 + T cells of seven participants, although at substantially lower levels that when corrected for the potential of contaminating memory CD4 + T cells, was still present in three. Near full-length proviral sequencing confirmed these findings across four participants. Our study findings are consistent with recent a recent study in children ages 5 – 11 years, where the mean contribution of naïve CD4 + T cells to the HIV-1 proviral pool was 13.5% [28]. In another study of children with HIV-1 in Thailand where the mean contribution was 17%, inducible and integrated HIV-1 was primarly located in memory CD4 + T cells even within the first five years of life [29]. More studies in adults have shown that naïve CD4 + T cells may also harbor intact proviruses capable of clonal expansion [19,28,41]. Such findings raise the possibility that pathways other than antigen-driven activation such as direct infection of naïve cells or resting CD4 + T cells contribute to the reservoir establishment [42–44]. It may be important to take account of proviruses in naïve cells as a reservoir in individuals living with perinatal HIV-1 infection in order to achieve a cure. Despite their low frequency, the proportion of intact proviruses tended to be higher in naïve CD4 + T cells than in memory cells. In studies of adults with non-perinatal HIV-1, naive CD4 + T cells have been shown to turn over less frequently and lead to slower decay of HIV-1 DNA [22], and may suggest that naïve CD4 + T cells serve as a more protected reservoir that is less prone to immune-mediated clearance or activation-induced proviral expression that requires consideration for their elimination. In the immature immune milieu of an infant, where naïve cells are the dominant CD4 + T cell subset, a small percentage of infected naïve cells could contribute substantially to the initial number of infected T cells, as shown in SHIV models in infant macaques [24]. However, studies are limited due to the larger blood volumes required for these studies.

Previous research and our results suggest that the paucity of naïve CD4 + T cells harboring proviral DNA after long-term ART is due to a combination of 1) the difficulty of viral entry into these cells and 2) a reduction in the proportion of naïve CD4 + T cells containing HIV-1 over time [28]. All env sequences were predicted to be CCR5 tropic, including in naïve CD4 + T cells, illustrating that infection of naïve CD4 + T cells did not reflect CXCR4-mediated entry, but likely occurred when naïve CD4 + T cells transiently express CCR5 [45] and activation markers such as HLA-DR [46]. The activation marker CD25, previously quantified in this cohort, correlated to proviral loads in both memory and naïve CD4 + T cells and offers additional evidence of an activated cellular state leading to infection and maintenance of proviral DNA. Our findings are consistent with both adult and perinatal HIV-1 studies, where R5-tropic HIV-1 was found in naïve CD4 + T cells [19,28]. In a previous study, we found the presence of CCR5 at very low levels on naïve CD4 + T cells of adolescents with perinatal HIV-1 [32], which may provide a sporadic window of opportunity for HIV-1 infection of naïve CD4 + T cells. HLA-DR was also detected at low levels (2.1%) in the naïve population and their expression was positively correlated with total HIV-1 DNA load [32]. The lack of compartmentalization of viruses in the naïve and memory CD4 + T cell, combined with the R5 tropic nature of viruses in the naïve cells, suggests that there are no inherent differences in the viruses that infect naïve CD4 + T cells. Rather, integration into naïve CD4 + T cells is a rare event that occurs when the cell becomes transiently permissive to HIV-1 infection and integration; this event may be more frequent in the case of perinatal HIV-1 due to the high frequency of naïve CD4 + T cells in infancy and young childhood [28,29]. In this cohort of AYA, however, the similarities between the sequences in the two T cell subsets may also arise due to the potential or naïve cells to repopulate the memory reservoir [19].

Integration site analysis revealed evidence of homeostatic proliferation in maintaining the viral reservoir in AYA with perinatal HIV-1, with nearly half of the integration sites detected in multiple cells. Integration was primarily in intronic regions of genes, with approximately half in the opposite orientation relative to the host gene, patterns consistent with non-perinatal adult reservoirs [47–49]. Prior studies suggest that proviruses oriented in the opposite direction of the host gene may have reduced expression due to transcriptional interference, and favor survival of the infected cells [50]. As AYA with perinatal HIV-1 maintain ART suppression, the proviral landscape also shifts, as can be seen in the correlations of age of virologic suppression to proviral load, and in the trend of larger clonal populations in those with longer duration of virologic suppression. Interestingly, HIV-1 DNA in naïve cells was highly correlated to CD4 + nadir, suggesting that proviral load in naïve CD4 + T cells may be influenced by immune reconstitution following ART.

Overall, analysis of the epigenetic landscape at site of integration reveals the preference for integration into activating marks and active genes, consistent with ease of integration into these sites. However, the trend of lower enrichment into regions with the activating mark H3K36me3 in our cohort with perinatal HIV-1 as compared to adults warrants further investigation. We also identified integrations into BACH2, STAT5B and ZNF gene families, sites previously associated with clonal expansion and persistence in adults with non-perinatal HIV-1 [37,49]. The similar patterns of integrations within these regions suggest shared mechanisms involved in proviral integration and maintenance between longstanding perinatal HIV-1 and non-perinatal HIV-1.

This study has several limitations. One is the small number of study participants and limited cell numbers, particularly for naïve subsets, and the use of an HIV-1 subtype B optimized IPDA for both subtype B and for the three participants with non-subtype B infection. The low abundance of HIV-1 in naïve cells increases the risk of potential biases due to contamination with memory CD4 + T cells, despite stringent gating. This potential contamination may also be present in both the sequences and integration sites detected in the naïve population. Thus, conclusions drawn from those datasets, while consistent with other published work, should be approached with caution. Additionally, integration site methods used here do not provide information on the intactness of integrated viral genome DNA, so the data includes proviruses that do not contribute to the replication-competent reservoir.

In summary, these findings show that the majority of the proviral reservoir resides in memory CD4 + T cells in AYA with perinatal HIV-1. Notably, naïve CD4 + T cells also contained persistent intact proviruses, which may be relatively resistant to reactivation and clearance, thereby making this pool of proviruses more challenging to target. Given that a child with perinatal HIV-1 is now expected to survive into the sixth and seventh decade of life [51], studies for ART-free remission and cure are imperative for this population. Our study findings of memory CD4 + T cells as the predominant reservoir in longstanding perinatal infection, coupled with the shared features of sites and orientation of viral integration and sequence structures and clonal expansion, as in adults with non-perinatal infection indicate that ART-free remission and cure strategies under development for adults with non-perinatal infection can be applied to this ever growing population of life-time survivors.

## Materials and methods

### Ethic statement

This study was approved by the Johns Hopkins Medicine Office of Human Subjects Research Institutional Review Boards as study number NA_00087629. Written informed consent was received from all participants before inclusion in the study.

### Study population

This research included a previously studied cohort of ten children and youths living with well-controlled HIV-1 for whom we reported on the size and magnitude of the induced reservoir [52]. The participants were recruited from the Johns Hopkins Pediatric Adolescent HIV/AIDS Program and the University of Maryland Division of Pediatric Immunology and Adolescent Medicine. The inclusion criteria were confirmed perinatal infection and effective suppression of viral replication to clinically undetectable plasma viral loads for one or more years; a single episode of intermittent low-level viremia (<400 copies/mL) was allowed. Duration of virologic suppression is calculated as the time between undetectable plasma viral load to blood draw; age at virologic suppression is calculated as age at which the latest period of virologic suppression occurred.

### Whole blood processing

Whole blood (35 – 50mL) was collected in EDTA tubes and processed within 24 hours of blood draw. PBMCs were isolated using Ficoll-Hypaque gradient centrifugation (GE Healthcare, Chicago, IL), treated with Red Blood Cell Lysis Buffer (eBioscience, San Diego, CA), and cryopreserved in FBS (Fetal Bovine Serum, Sigma Aldrich) containing 10% DMSO. For the following assays, PBMCs were thawed in 50% FBS and 50% RPMI 1640 and rested overnight at 37°C in complete media (RPMI 1640 with Glutamax, with 10% FBS and 1x penicillin/streptomycin).

### Flow cytometry analyses of T cell populations and Sorting for Naïve and Memory CD4 + T cells

One million cryopreserved PBMCs were thawed and rested overnight. The following day, the cells were resuspended and washed with PBS in 5ml round bottom tubes before staining with the following antibodies: CD3 APC-R700, CD4 BV711, CD45RA APC, CCR7 BB700, CD28 BUV805, CCR5 BV650 (BD Biosciences). Isotype controls were added to corresponding Fluorescence Minus One (FMO) controls at the same concentration as the corresponding antibody. All tubes were incubated at room temperature in the dark for 30 minutes. Cells were then washed with stain buffer (PBS with 1% FBS) and fix/permed with Foxp3/Transcription Factor Staining Buffer Set (eBioscience). After the final incubation, cells were washed twice with Permeabilization Buffer and resuspended in 200 µl stain buffer, stored away from light at 4 °C on ice for analysis the next day on a Symphony A3 flow cytometer using FACSDiva software version 10 (BD Biosciences) through the Johns Hopkins Bloomberg Flow Cytometry and Immunology Core. Cell phenotypic data were analyzed using FlowJo software (version 10.2, Tree Star). Gating was defined using fluorescent minus one and isotype controls.

### Cell sorting

70 million cryopreserved PBMCs were thawed and rested overnight. The next day, cells were placed in complete media at 10 million cells per mL and stored at 4°C for 2 hours, following which CD4 + T cells were enriched with a negative enrichment system (Miltenyi Biotec, Bergisch Gladbach, Germany). The cells were stained with the following antibodies: CD3 FITC, CD4 PE, CCR7 BB700, CD45RA APC, CD95 BV421 (BD Biosciences), along with propidium iodide for viability to allow exclusion of dead cells. Cells were sorted using a BD FACS Aria III Cell Sorter as naïve CD4 + T cells through two gatings of (CD3+ , CD4+ , CCR7+ , CD45RA+) cells, and (CD43 + , CD4 + , CD95-). The remaining CD3+ , CD4+ were categorized as memory CD4+ T cells. A purity check was performed on a subset of sorted naïve CD4+ T cells and quantified for contaminating memory CD4 + T cells. Following cell sorting, cells were pelleted and lysed with Gentra Cell Lysis Buffer (Qiagen, Germantown, MD) and the lysed cells were stored at room temperature.

### HIV-1 DNA quantification

Genomic DNA was isolated from one to six million naïve and memory CD4 + T cell subsets using the Gentra Puregene kit (QIAGEN, Germantown, MD). DNA was eluted in 50µL of elution buffer, then quantified with a Qubit High Sensitivity Kit. HIV-1 DNA was quantified using the multiplex droplet digital polymerase chain reaction (ddPCR) Intact Proviral DNA Assay (IPDA) [10]. The IPDA allows for the simultaneous detection of HIV-1 Ψ and HIV-1 ENV in a given provirus, which further allows for quantification of intact and defective proviruses in the analyzed T cell subsets. A total of 1.6 µg of DNA was assayed for each sample tested and carried out in either replicates of 8 or 16 based on the concentration of genomic DNA isolated from each T cell subset. Droplets were generated using a QX200 Droplet Generator and the PCR reaction was performed according to these temperatures: 95°C for 10 min, 60 cycles of 94°C for 30 sec and 53°C for 60 sec, 98°C for 10 min, and a final overnight rest at 12°C. Droplets were read with a QX200 Droplet Reader. The human gene RPP30 (Table 2) was used to estimate both the number of cells analyzed and, with two primer and probe sets as previously reported, to allow estimation and correction for DNA shearing during processing. Proviral load was determined as copies per million CD4 + T cells and by intact, 5’ or 3’ defectives, along with estimated total proviral load as the sum of the three species.

**Table 2:** List of primers and probes for multiplexed ddPCR and sequencing.

Primer/Probe Name	Sequence
GAG Fw	TCTCGACGCAGGACTCG
GAG Rv	TACTGACGCTCTCGCACC
GAG Probe	/56-FAM/CTCTCTCCT/ZEN/TCTAGCCTC/3IABkFQ/
Env Fw	AGTGGTGCAGAGAGAAAAAAGAGC
Env Rv	GTCTGGCCTGTACCGTCAGC
Env Probe	/5HEX/CCTTGGGTT/ZEN/CTTGGGAGC/3IABkFQ/
Hypermut Probe	/5IABkFQ/CCTTAGGTTCTTAGGAGC/3IABkFQ/
RPP30 Shear Fw	CCATTTGCTGCTCCTTGGG
RPP30 Shear Rv	CATGCAAAGGAGGAAGCCG
RPP30 Shear Probe	/56-FAM/AAGGAGCAA/ZEN/GGTTCTATTGTAG/3IABkFQ/
RPP30 Fw	GATTTGGACCTGCGAGCG
RPP30 Rv	GCGGCTGTCTCCACAAGT
RPP30 Probe	/5HEX/CTGACCTGA/ZEN/AGGCTCT/3IABkFQ/
BLOuterF	AAA TCT CTA GCA GTG GCG CCC GAA CAG
BLOuterR	TGA GGG ATC TCT AGT TAC CAG AGT C
U5-638F	GCGCCCGAACAGGGACYTGAAARCGAAAG
BLInnerR	GCACTCAAGGCAAGCTTTATTGAGGCTTA

### Limiting dilution near full length HIV-1 sequencing

Purified DNA was plated in 40 wells at limiting dilution, such that 30% or fewer wells contained proviral DNA. The near full length (nFL) proviral genome of approximately 9kb was amplified with a LongAmp Taq (New England Biosystems, Ipswitch MA) with the primers BLOuterF and BLOuterR shown in Table 2, with the following PCR conditions: 94°C for 2m, 3 cycles of (94°C for 30s, 64°C for 30s, 65°C for 10m), 3 cycles of (94°C for 30s, 61°C for 30s, 65°C for 10m), 3 cycles of (94°C for 30s, 58°C for 30s, 65°C for 10m), 21 cycles of (94°C for 30s, 55°C for 30s, 65°C for 10m), Final extension at 65°C for 10m. Following this initial PCR, the samples were diluted 1:50, then an inner nested 9kb amplicon was amplified with the LongAmp Taq kit with the primers U5-638F and BLInnerR shown in Table 2, with the following PCR conditions: 94°C for 2m, 3 cycles of (94°C for 30s, 64°C for 30s, 65°C for 10m), 3 cycles of (94°C for 30s, 61°C for 30s, 65°C for 10m), 3 cycles of (94°C for 30s, 58°C for 30s, 65°C for 10m), 36 cycles of (94°C for 30s, 55°C for 30s, 65°C for 10m), Final extension at 65°C for 10m. Gel electrophoresis was performed to determine the presence of HIV-1 DNA, and was submitted to the Massachusetts General Hospital Center for Computation and Integrative Biology. Sequences were aligned against HXB2 and assessed for intactness using the HIVSeqinR pipeline [53,54]. Phylogenic trees were generated using maximum likelihood estimates in MEGA X [55]. Tropism of proviruses was determined using WebPSSM [34].

### Integration site analysis by the SonicAbundance method

Integration site analysis was performed by the Viral Molecular High Density Sequencing Core at the University of Pennsylvania. Analysis of integration site distributions was carried out using ligation-mediated PCR as described [35,36]. Genomic DNA samples were sheared by sonication, and DNA adaptors ligated to the free DNA ends. Two rounds of PCR were then used to amplify the genomic segment between the HIV LTR and the adaptor. Sequence data was acquired from both the LTR side and the adaptor side of each DNA molecule using the Illumina method. Genomic sequence was then mapped onto the human genome (vHS1/T2T-CHM13 draft genome). Sequence data was processed and quality controlled using previously described methods [35,36] and further at https://github.com/helixscript/AAVengeR. Clone size was quantified using the sonicAbundance method [56], in which the number of linker ligation sites is used as a proxy for the number of independent DNA chains sampled that represent each integration site. Intact and env sequences used in this study are deposited in GenBank under accession numbers **PV691810-PV691828**; integration site data are deposited at the NCBI SRA under accession number **PRJNA1263566** (S1 Table).

### Gene ontology over representation analysis

Over-represented pathways in the Gene Ontology (GO) categories of biological processes, cellular components, and molecular functions were obtained using the clusterProfiler package using the enrichGo() method [57]. All genes were considered for analysis, and annotations were obtained from the AnnotationDbi package [58] b. Significantly overrepresented pathways were selected using a significance threshold of <0.05 after p value adjustment for multiple comparisons.

### Adult HIV integration analyses

Raw sequencing reads containing integration site data from 13 participants of acute, chronic, or ART-treated HIV status from a previously published study were obtained and processed using AAVengeR as described in the previous section [59].

### Genomic and epigenetic feature correlation and comparative analyses

Integration sites were annotated for presence in transcription units and the start of transcription units using RefSeq annotations obtained from UCSC (hgdownload.soe.ucsc.edu/gbdb/hs1/ncbiRefSeq), within ENCODE candidate Cis-Regulatory Elements cCREs, within epigenetic CHIP-seq tracks from ENCODE, and within repeats obtained by RepeatMasker [60–63]*.* GC content at varying window sizes surrounding an integration were extracted from the HS1/T2T genome. For each sample, integration distributions at all features were calculated at varying window sizes and compared to a random distribution of integration sites three times the size of the sample to obtain the receiver operator characteristic (ROC) using the hotROCs package (https://github.com/BushmanLab/hotROCs) and implemented in the InvestigateIntegrations package (https://github.com/agmcfarland/InvestigateIntegrations) [36]. Track coordinates originally available in using hg38 assembly were converted to HS1/T2T coordinates using liftover [64]. For every feature and window size, ROCs greater than 0.5 indicates more integrations compared to a random distribution model while ROCs less than 0.5 indicates fewer integrations.

Integration site distributions between adult and AYA participants from this study was performed by combining integration sites from all cell types per participant followed by annotation and ROC calculation. Multiple comparison correction was performed over all participant data.

### Statistical methods

Comparisons were performed using nonparametric tests due to small sample sizes. Wilcoxon’s signed rank tests were used for paired tests, and Mann-Whitney rank-sum tests were used for unpaired tests. Spearman Rank test was used to determine correlations to demographics and previously published activation and exhaustion in the same cohort. Significance was established at p < 0.05. The potential contamination of memory cells in the naïve population was calculated based on a 98% purity of the naïve cell sort, and a lower estimate of infected naïve cells was calculated at a 95% confidence interval based on previously published methods [28]. The base R method stats:: cor.test (method = ‘spearman’) was used for correlations between longitudinal data and integration site metrics, P-value correction was performed using the Benjamini-Hochberg method using the base R package method stats::p.adjust().

## Supporting information

S1 Table Mean and maximal contribution of memory cell contamination on inferred intact HIV-1 DNA detected in naïve CD4+ T cell sorted population. (DOCX)

S2 Table Mean and maximal contribution of memory cell contamination on total HIV-1 DNA detected in naïve CD4+ T cell sorted population. (DOCX)

S3 Table Integration sites identified and associated annotation. Note that only unique integration sites are shown, and not integration sites in repeated sequences that did not map uniquely on the human genome.
(CSV)

S1 Fig Percentage of Naïve CD4+ T cells (Tn, CD3+ CD4+ CCR7+ CD45RA+ CD28+, CD95), Central memory (Tcm, CD3+ CD4+ CCR7+ CD45RA- CD28+), and Transitional (CD3+ CD4+ CCR7- CD45RA- CD28+) + Effector (CD3+ CD4+ CCR7- CD45RA- CD28-, Ttem) memory cells. (DOCX)

S2 Fig Gating strategy for cell sorting. A) Naïve cells from participant 0117 are sorted as CD3+ CD4+ CCR7+ CD45RA+, with an an additional gating of CD3+ CD4+ CD95- to remove stem cell-like memory T cells. A gate for memory cells was used which combines central memory, effector memory, transitional memory and TEMRA CD4+ T cells. B) A purity check was performed in a subset of participants, where sorted naïve cells were re-assessed for contaminated memory cells, which was estimated to be 2% of sorted naïve cells, predominantly CD45RA+ CCR7- TEMRA.
(DOCX)

S3 Fig Correlations of Intact and Total HIV-1 DNA in memory and naïve CD4+ T cells to immune activation and exhaustion markers. Correlations were determined using Spearman Rank.
(DOCX)

S4 Fig Combined integration sites detected within gene coding regions from all participants. Top 10 genes with the highest number of integration sites, with number of integration sites are shown.
(DOCX)

S5 Fig Correlation duration of virologic suppression and age at virologic suppression to % singleton (unique integration sites only detected once) of total integration sites detected by participant. Correlation was calculated using Spearman Rank.
(DOCX)

S6 Fig Comparison of perinatal and adult integration sites and enrichment in A) activation and B) repressive epigenetic markers. For each sample, the number of integration sites within each feature and window size are compared to a random distribution to generate an ROC. An ROC greater than 0.5 indicates a sample’s integration sites occur within the given feature and window size at a higher rate compared to random while an ROC less than 0.5 indicates a rate lower than random. Window sizes show degree of expansion of track boundaries. A window size of zero uses unchanged track bounds while increasing sizes expand the start and ends of tracks by half the displayed amount. Each dot shows a single sample’s ROC. For each group, lines show the mean and 95% confidence interval of the ROC. Adult samples are separated by participant HIV status (acute, ART-treated, chronic). AYA samples from this study are indicated by JH. ROC: receiver operator characteristic. Act: dataset derived from activated T cells.
(DOCX)

S7 Fig Gene ontology overrepresentation analysis of integration sites, separated into molecular functions (red), biological processes (blue) and cellular components (purple). The gene ratio is the ratio of genes with a given GO term containing an integration site divided by the total number of genes with an integration site. A) Analysis of combined integration sites within memory, naïve and total CD4+ T cells. B) Analysis of integration sites within naïve CD4+ T cells. Adjusted p-values are shown, *: p < 0.05, **: p < 0.01.
(DOCX)
