# Supplementary material for: Quantification and localization of integrated HIV-1 in memory and naïve CD4+ T cells from adolescents and young adults with perinatally-acquired HIV-1
Source: PLoS Pathog. 2026 Jul 13;22(7):e1014369. doi: 10.1371/journal.ppat.1014369 (PMC13399508; doi:10.1371/journal.ppat.1014369)
Supplement: S1 Table — (DOCX) [file ppat.1014369.s001.docx]

**Supplementary Table 1:** Mean and maximal contribution of memory cell contamination on inferred intact HIV-1 DNA detected in naïve CD4+ T cell sorted population.

| **Participant ID** | **HIV-infected cells/10^6^ cells in naïve cells** | **Maximum no. HIV-infected memory cells/10^6^ cells in naïve sort** | **Expected no. of HIV-infected memory cells/10^6^ cells in naïve sort** | **Minimum no. of HIV-infected naïve cells/10^6^ cells in naïve sort** | **Expected no. of HIV-infected naïve cells/10^6^ cells in naïve cell sort** |
| --- | --- | --- | --- | --- | --- |
| 0117 | 12.2 | 6.9 | 3.6 | 5.3 | 8.6 |
| 0301 | 23.2 | 2.8 | 1.6 | 20.4 | 21.5 |
| 0307 | 1.5 | 28.6 | 25.9 | <1 | <1 |
| M0105 | 4.1 | 3.7 | 2.0 | <1 | 2.1 |
