# Supplementary material for: Quantification and localization of integrated HIV-1 in memory and naïve CD4+ T cells from adolescents and young adults with perinatally-acquired HIV-1
Source: PLoS Pathog. 2026 Jul 13;22(7):e1014369. doi: 10.1371/journal.ppat.1014369 (PMC13399508; doi:10.1371/journal.ppat.1014369)
Supplement: S2 Table — (DOCX) [file ppat.1014369.s002.docx]

**Supplementary Table 2:** Mean and maximal contribution of memory cell contamination on total HIV-1 DNA detected in naïve CD4+ T cell sorted population.

| **Participant ID** | **HIV-infected cells/10^6^ cells in naïve cells** | **Maximum no. HIV-infected memory cells/10^6^ cells in naïve sort** | **Expected no. of HIV-infected memory cells/10^6^ cells in naïve sort** | **Minimum no. of HIV-infected naïve cells/10^6^ cells in naïve sort** | **Expected no. of HIV-infected naïve cells/10^6^ cells in naïve cell sort** |
| --- | --- | --- | --- | --- | --- |
| 0117 | 43.2 | 53.9 | 44.7 | <1 | <1 |
| 0300 | 94.0 | 5.2 | 3.8 | 88.8 | 90.2 |
| 0301 | 116.7 | 66.8 | 58.3 | 49.8 | 58.3 |
| 0304 | 82.0 | 49.8 | 42.7 | 32.2 | 39.2 |
| 0305 | 7.0 | 42.6 | 35.8 | <1 | <1 |
| 0307 | 16.7 | 181.6 | 164.9 | <1 | <1 |
| M0105 | 17.9 | 39.8 | 30.8 | <1 | <1 |
