# Supplementary material for: Quantification and localization of integrated HIV-1 in memory and naïve CD4+ T cells from adolescents and young adults with perinatally-acquired HIV-1
Source: PLoS Pathog. 2026 Jul 13;22(7):e1014369. doi: 10.1371/journal.ppat.1014369 (PMC13399508; doi:10.1371/journal.ppat.1014369)
Supplement: S1 Fig — (DOCX) [file ppat.1014369.s004.docx]

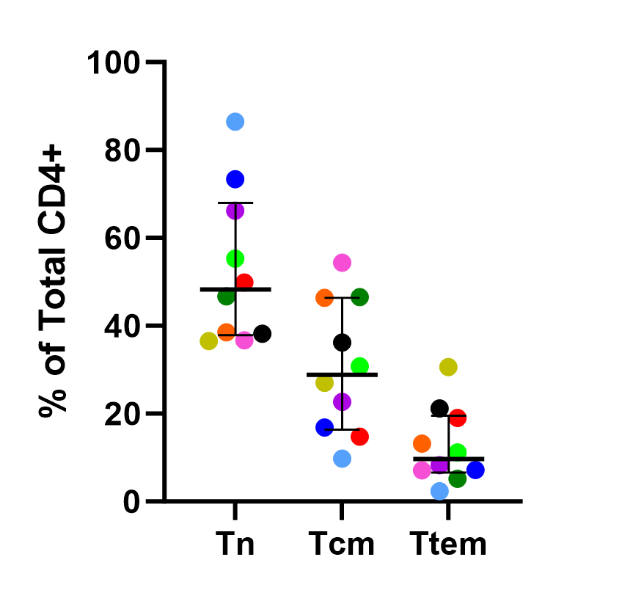

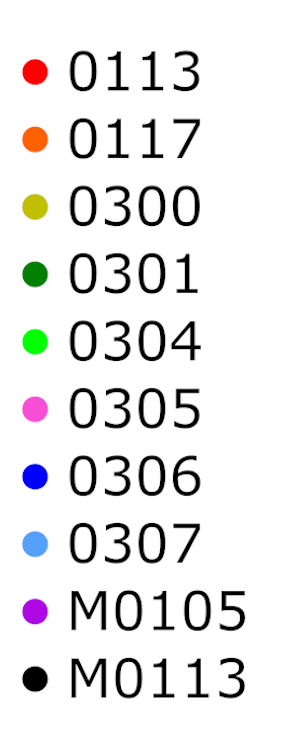


**Supplemental Figure 1**: Percentage of Naïve CD4+ T cells (Tn, CD3+ CD4+ CCR7+ CD45RA+ CD28+, CD95), Central memory (Tcm, CD3+ CD4+ CCR7+ CD45RA- CD28+), and Transitional (CD3+ CD4+ CCR7- CD45RA- CD28+) + Effector (CD3+ CD4+ CCR7- CD45RA- CD28-, Ttem) memory cells.
