# Supplementary material for: Quantification and localization of integrated HIV-1 in memory and naïve CD4+ T cells from adolescents and young adults with perinatally-acquired HIV-1
Source: PLoS Pathog. 2026 Jul 13;22(7):e1014369. doi: 10.1371/journal.ppat.1014369 (PMC13399508; doi:10.1371/journal.ppat.1014369)
Supplement: S2 Fig — A) Naïve cells from participant 0117 are sorted as CD3+ CD4+ CCR7+ CD45RA+, with an an additional gating of CD3+ CD4+ CD95- to remove stem cell-like memory T cells. A gate for memory cells was used which combines central memory, effector memory, transitional memory and TEMRA CD4+ T cells. B) A purity check was performed in a subset of participants, where sorted naïve cells were re-assessed for contaminated memory cells, which was estimated to be 2% of sorted naïve cells, predominantly CD45RA+ CCR7- TEMRA. (DOCX) [file ppat.1014369.s005.docx]

**A**

**
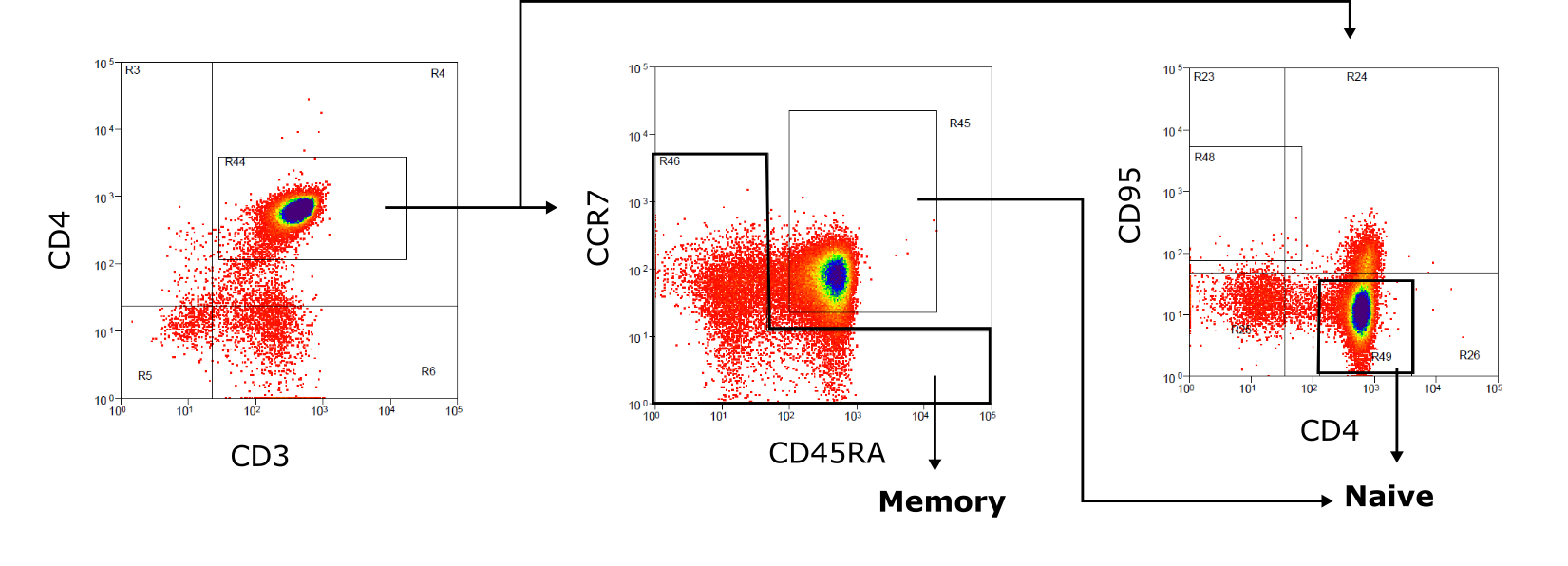
**

**B**


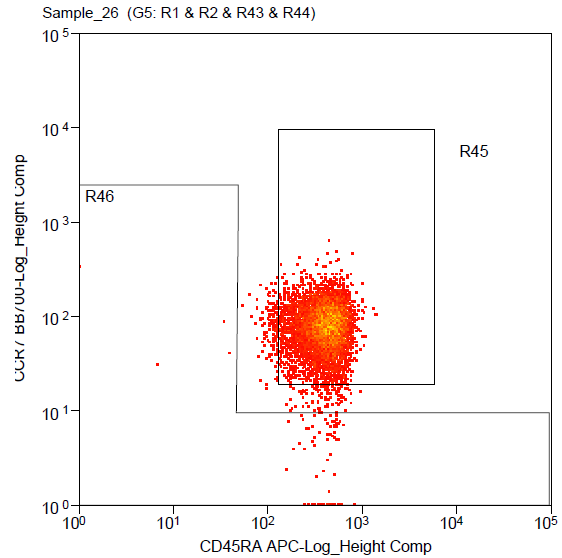


**Supplemental Figure 2: Gating strategy for cell sorting.** A) Naïve cells from participant 0117 are sorted as CD3+ CD4+ CCR7+ CD45RA+, with an an additional gating of CD3+ CD4+ CD95- to remove stem cell-like memory T cells. A gate for memory cells was used which combines central memory, effector memory, transitional memory and TEMRA CD4+ T cells. B) A purity check was performed in a subset of participants, where sorted naïve cells were re-assessed for contaminated memory cells, which was estimated to be 2% of sorted naïve cells, predominantly CD45RA+ CCR7- TEMRA.
