# Supplementary material for: Quantification and localization of integrated HIV-1 in memory and naïve CD4+ T cells from adolescents and young adults with perinatally-acquired HIV-1
Source: PLoS Pathog. 2026 Jul 13;22(7):e1014369. doi: 10.1371/journal.ppat.1014369 (PMC13399508; doi:10.1371/journal.ppat.1014369)
Supplement: S3 Fig — Correlations were determined using Spearman Rank. (DOCX) [file ppat.1014369.s006.docx]

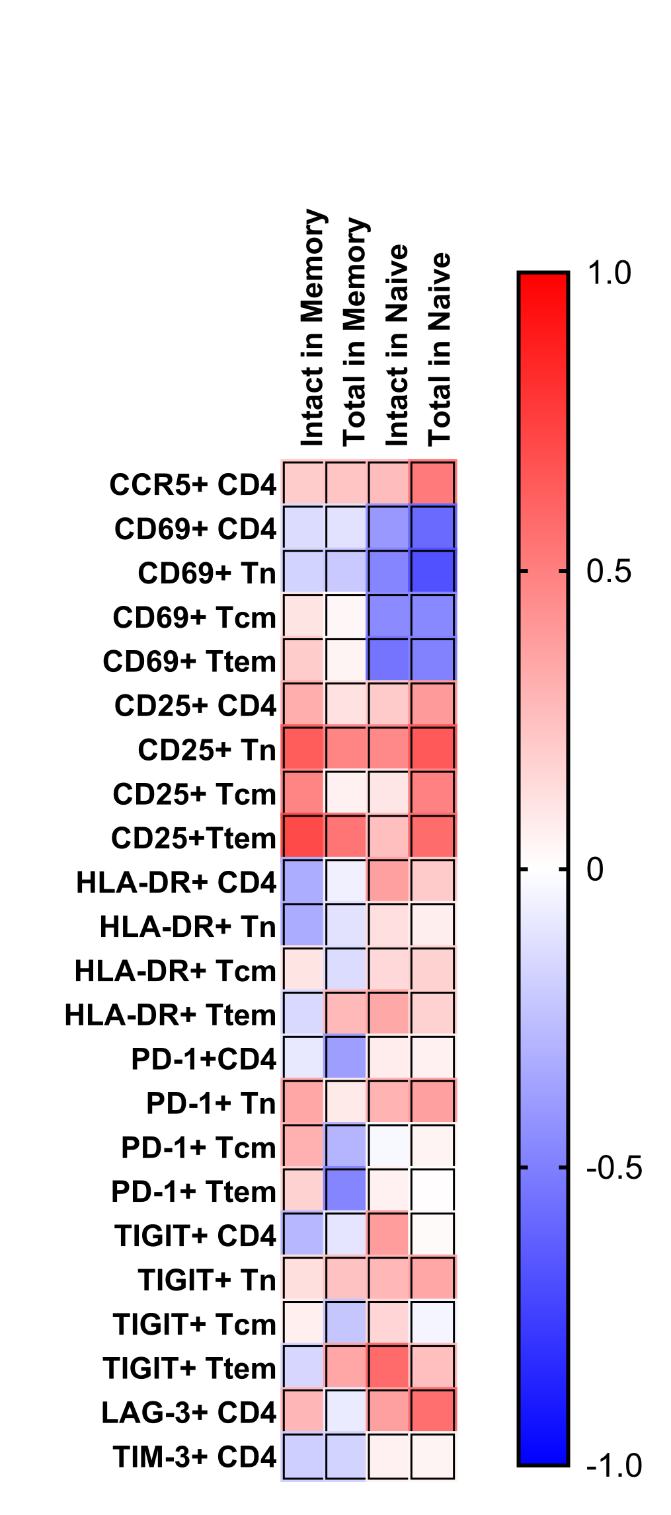


**Supplemental Figure 3: Correlations of Intact and Total HIV-1 DNA in memory and naïve CD4+ T cells to immune activation and exhaustion markers.** Correlations were determined using Spearman Rank.
