# Supplementary material for: Quantification and localization of integrated HIV-1 in memory and naïve CD4+ T cells from adolescents and young adults with perinatally-acquired HIV-1
Source: PLoS Pathog. 2026 Jul 13;22(7):e1014369. doi: 10.1371/journal.ppat.1014369 (PMC13399508; doi:10.1371/journal.ppat.1014369)
Supplement: S4 Fig — Top 10 genes with the highest number of integration sites, with number of integration sites are shown. (DOCX) [file ppat.1014369.s007.docx]

**
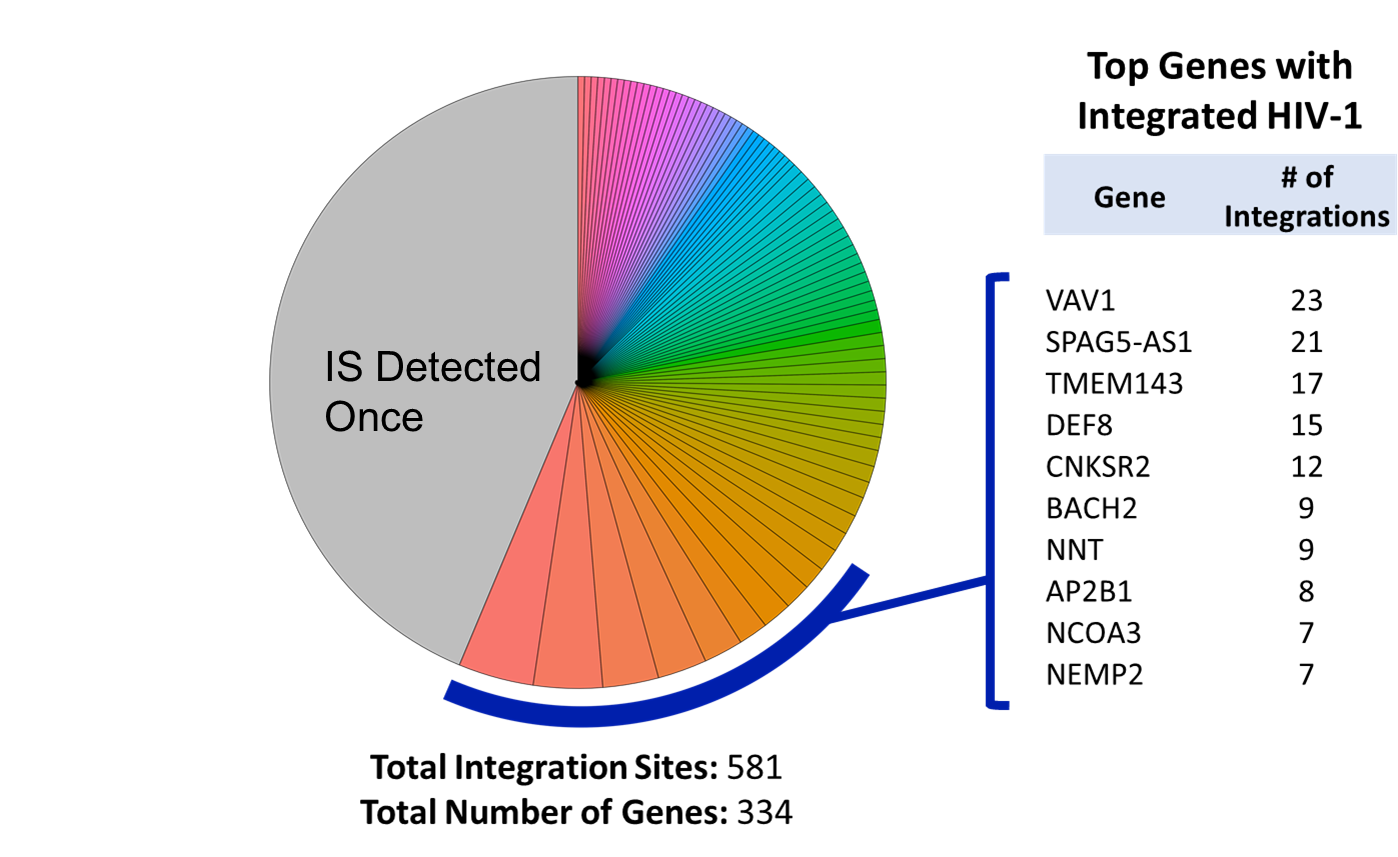
**

**Supplementary Figure 4: combined integration sites detected within gene coding regions from all participants**. Top 10 genes with the highest number of integration sites, with number of integration sites are shown.
