# Supplementary material for: Quantification and localization of integrated HIV-1 in memory and naïve CD4+ T cells from adolescents and young adults with perinatally-acquired HIV-1
Source: PLoS Pathog. 2026 Jul 13;22(7):e1014369. doi: 10.1371/journal.ppat.1014369 (PMC13399508; doi:10.1371/journal.ppat.1014369)
Supplement: S5 Fig — Correlation was calculated using Spearman Rank. (DOCX) [file ppat.1014369.s008.docx]

**
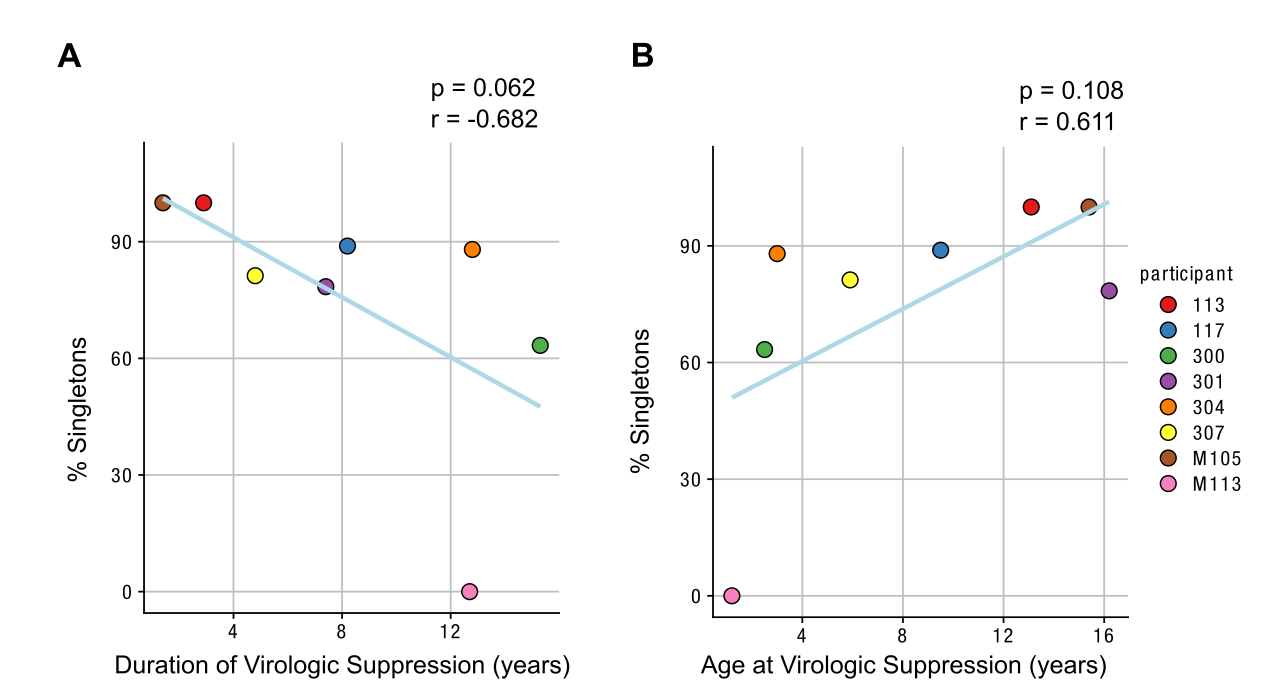
**

**Supplemental Figure 5: Correlation duration of virologic suppression and age at virologic suppression to % singleton (unique integration sites only detected once) of total integration sites detected by participant.** Correlation was calculated using Spearman Rank.
