# Supplementary material for: Quantification and localization of integrated HIV-1 in memory and naïve CD4+ T cells from adolescents and young adults with perinatally-acquired HIV-1
Source: PLoS Pathog. 2026 Jul 13;22(7):e1014369. doi: 10.1371/journal.ppat.1014369 (PMC13399508; doi:10.1371/journal.ppat.1014369)
Supplement: S6 Fig — For each sample, the number of integration sites within each feature and window size are compared to a random distribution to generate an ROC. An ROC greater than 0.5 indicates a sample’s integration sites occur within the given feature and window size at a higher rate compared to random while an ROC less than 0.5 indicates a rate lower than random. Window sizes show degree of expansion of track boundaries. A window size of zero uses unchanged track bounds while increasing sizes expand the start and ends of tracks by half the displayed amount. Each dot shows a single sample’s ROC. For each group, lines show the mean and 95% confidence interval of the ROC. Adult samples are separated by participant HIV status (acute, ART-treated, chronic). AYA samples from this study are indicated by JH. ROC: receiver operator characteristic. Act: dataset derived from activated T cells. (DOCX) [file ppat.1014369.s009.docx]

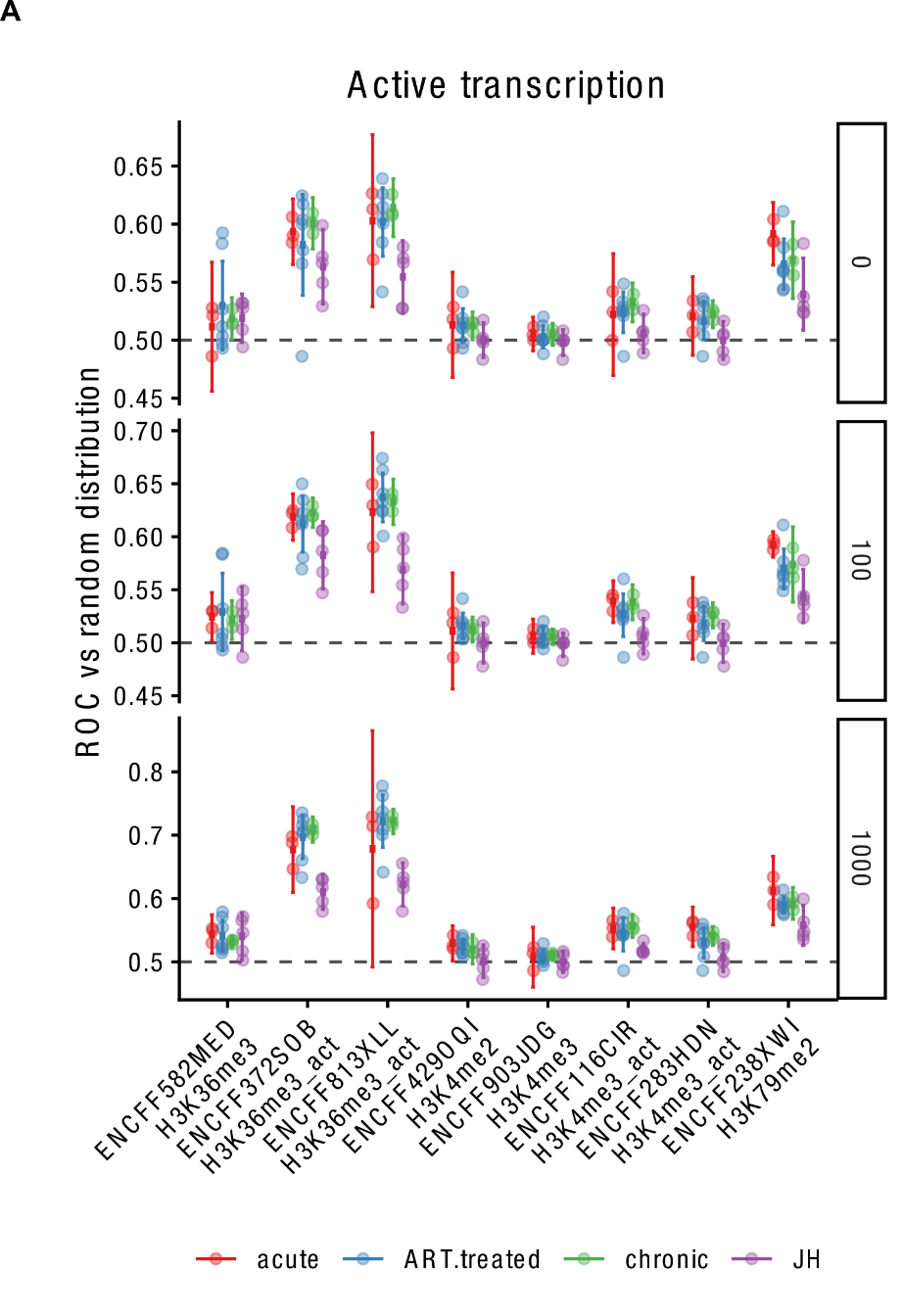


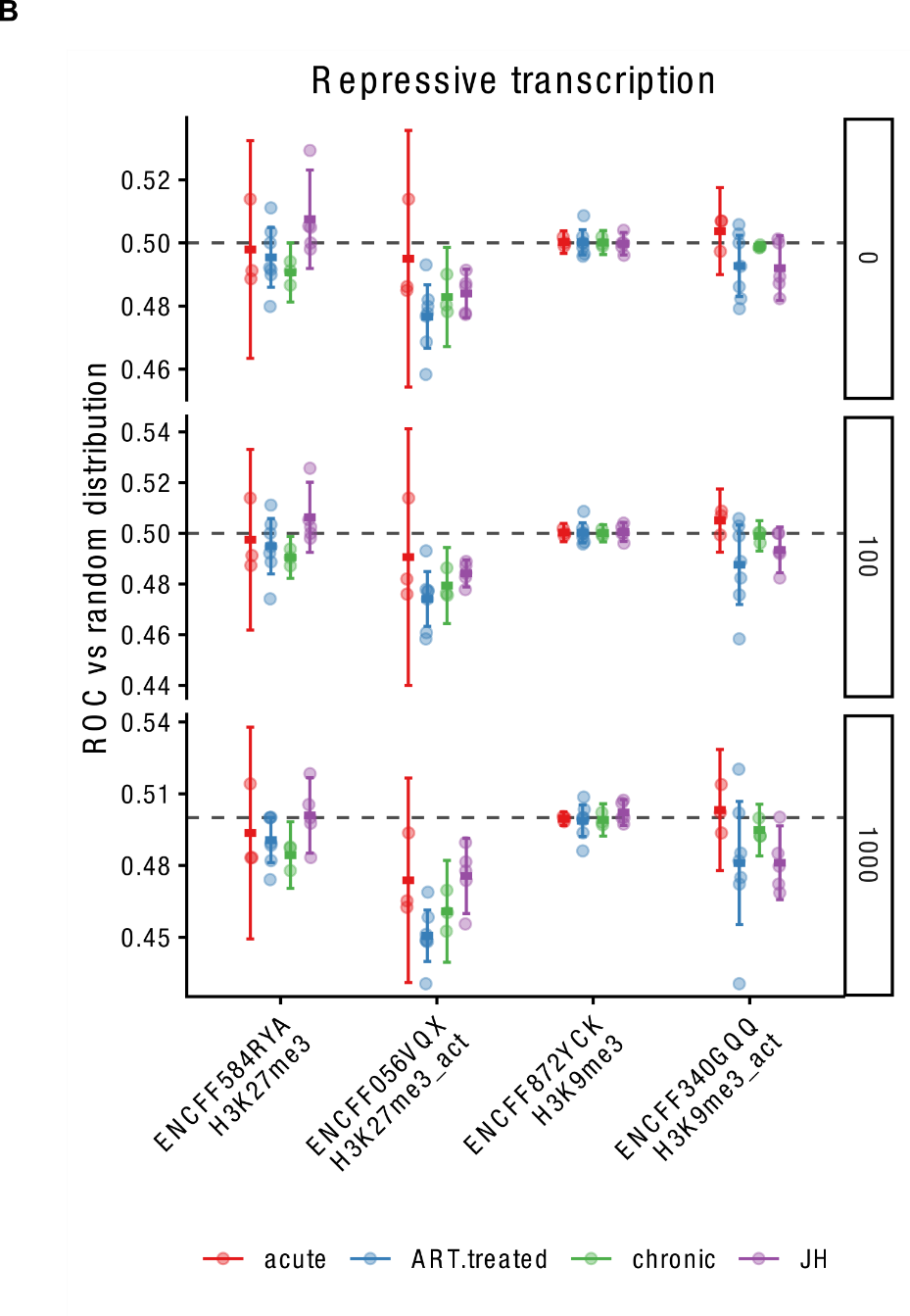


**Supplemental Figure 6: Comparison of perinatal and adult integration sites and enrichment in A) activation and B) repressive epigenetic markers.** For each sample, the number of integration sites within each feature and window size are compared to a random distribution to generate an ROC. An ROC greater than 0.5 indicates a sample’s integration sites occur within the given feature and window size at a higher rate compared to random while an ROC less than 0.5 indicates a rate lower than random. Window sizes show degree of expansion of track boundaries. A window size of zero uses unchanged track bounds while increasing sizes expand the start and ends of tracks by half the displayed amount. Each dot shows a single sample’s ROC. For each group, lines show the mean and 95% confidence interval of the ROC. Adult samples are separated by participant HIV status (acute, ART-treated, chronic). AYA samples from this study are indicated by JH. ROC: receiver operator characteristic. Act: dataset derived from activated T cells.
