# Supplementary material for: Quantification and localization of integrated HIV-1 in memory and naïve CD4+ T cells from adolescents and young adults with perinatally-acquired HIV-1
Source: PLoS Pathog. 2026 Jul 13;22(7):e1014369. doi: 10.1371/journal.ppat.1014369 (PMC13399508; doi:10.1371/journal.ppat.1014369)
Supplement: S7 Fig — The gene ratio is the ratio of genes with a given GO term containing an integration site divided by the total number of genes with an integration site. A) Analysis of combined integration sites within memory, naïve and total CD4+ T cells. B) Analysis of integration sites within naïve CD4+ T cells. Adjusted p-values are shown, *: p < 0.05, **: p < 0.01. (DOCX) [file ppat.1014369.s010.docx]

**
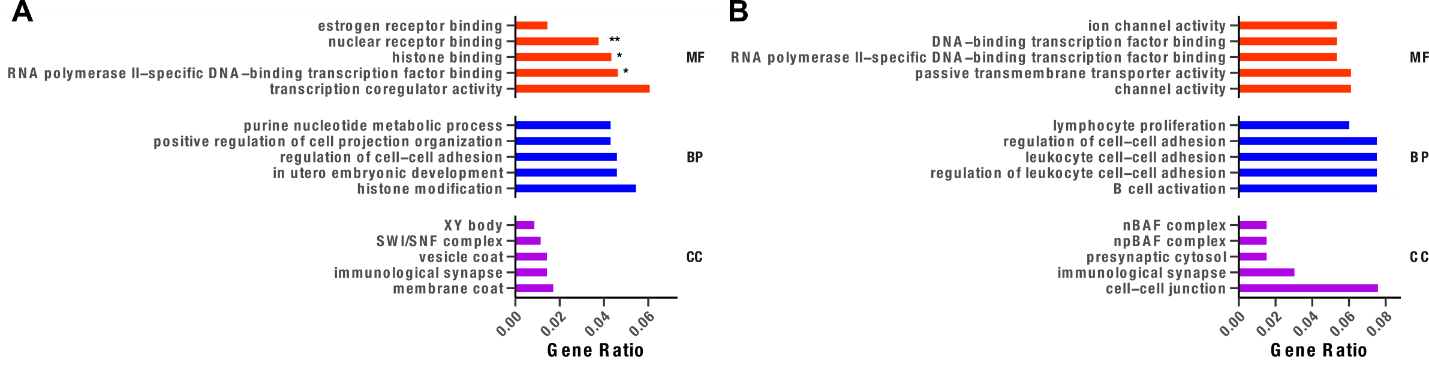
**

**Supplemental Figure 7:** Gene ontology overrepresentation analysis of integration sites, separated into molecular functions (red), biological processes (blue) and cellular components (purple). The gene ratio is the ratio of genes with a given GO term containing an integration site divided by the total number of genes with an integration site. A) Analysis of combined integration sites within memory, naïve and total CD4+ T cells. B) Analysis of integration sites within naïve CD4+ T cells. Adjusted p-values are shown, *: p<0.05, **: p<0.01.
