# Supplementary material for: Quantification and localization of integrated HIV-1 in memory and naïve CD4+ T cells from adolescents and young adults with perinatally-acquired HIV-1
Source: PLoS Pathog. 2026 Jul 13;22(7):e1014369. doi: 10.1371/journal.ppat.1014369 (PMC13399508; doi:10.1371/journal.ppat.1014369)
Supplement: Attachment — Submitted filename: Reviewer comment responses 20260504.docx [file ppat.1014369.s012.docx]

Dear Dr. Kearney and Editors,

Thank you for reviewing our manuscript, entitled “Quantification and localization of integrated HIV-1 in memory and naïve CD4+ T cells from adolescents and young adults with perinatally-acquired HIV-1”, for consideration for publication in *Plos Pathogens*. We appreciate the opportunity to strengthen our manuscript in response to the editors’ reviewers’ comments. We have addressed the critiques and believe these critiques have resulted in a stronger manuscript with the suggested new analyses. Please find our responses to the reviewers’ comments:

Reviewer #1: The significance of the work would be greatly enhanced by uncovering some new insight from existing data or through additional experimentation - probably the latter. Correlative integration site/gene-expression analysis or matching intact proviruses to sites of integration might serve in this respect, though this cannot be predicted with certainly and will no doubt be made more challenging by the demonstrated difficulty in finding and characterizing naive T cell integration sites in these samples.

- **Unfortunately, there are no remnant samples at the visits presented in this paper, preventing additional experimentation. However, we have performed additional analyses as suggested, including additional correlations with immune activation and exhaustion from the same cohort in a previous publication (Huang et al., Front Immunol. 2023, lines 210-215). We have also done additional analyses of the integration site data, for correlations to published datasets on epigenetic marks (lines 280-286, Figure 6), and comparisons of integration sites in adult cohorts (lines 286-291, Supplemental Figure 6)**

Moreover, since proviral persistence in naive CD4+ T cells is one of the principal findings of this work, the possibility that apparently infected naive cells are actually contaminated memory cells must be more convincingly ruled out by statistical analysis that is more rigorous that that provided in Results. Specifically, the authors should calculate 95% confidence intervals for naive T cell infection frequencies based on sort purities, memory cell infection frequencies, and other factors where measured frequencies greater that this interval cannot reasonably be explained by infected memory cell contamination alone. Details for performing this statistical analysis are provided in Katusiime, et al.

- **We thank the reviewer for suggesting the approach to exclude the potential for contaminating memory CD4+ T cells as contributing to the detection of proviral persistence in naïve CD4+ T cells. The 95% confidence interval was calculated for both the intact and total HIV-1 DNA loads (Supplemental Tables 1 and 2). The data has been changed from a graph summarizing the means to a table that includes both the mean and 95% confidence intervals estimated memory cell contamination. Even after the correction, the results and conclusions remain the same: with three participants having total and two participants having intact proviral loads quantified in the naïve CD4+ T cell population, which cannot be explained by memory cell contamination alone (Lines 180-195).**

Reviewer #1: Because ART suppresses ongoing HIV replication, samples taken even after years on ART to some extent reflect the state of the infection immediately prior to ART administration, in this case in an immune environment in which the fraction of CD4+ T cells exceed 90% at birth but declines rapidly with age. Therefore, participant ages at the time of initial ART administration and at the first test of undetectable viremia should be added to Table 1. This would, for example, enable the authors to potentially establish a more granular correlation between participant age at initial viremic suppression and naive T cell contribution to the pool of infected T cells at the time of sampling, which would strengthen the work considerably.

- **Due to the nature of the participants who are recruited at Johns Hopkins and the University of Maryland, the age at ART initiation and age at initial virologic suppression are unknown in many of the individuals. As a proxy, we have included the age at virologic suppression as determined by the inclusion criteria (confirmed perinatal infection and effective suppression of viral replication to clinically undetectable plasma viral loads for one or more years; a single episode of intermittent low-level viremia was allowed, Table 1). Many of these individuals started ART early, but have extended periods of ART non-adherence, which we believe play a role in shaping the reservoir. Thus, we have added additional analyses correlating both inferred intact and total copies by IPDA, as well as correlations to integration sites.**

Please refer to IPDA+ proviruses as "potentially intact", "inferred intact", or similar, rather than "intact". "Intact" or "genetically intact" should only refer to proviruses that have been sequenced and verified as capable of producing new virus by some established standard. Intact proviral sequences should also be submitted to GenBank.

- **Analysis and quantification of the double positive droplets from the ddPCR assay has now been changed to “inferred intact” throughout. Any data generated from sequencing has been kept as intact, and these sequences have already been uploaded to GenBank under the accession numbers PV691810-PV691828.**

Phylogenetic co-mingling of env sequences from naive and memory cells sorts may be a result of memory cell contamination. This possibility should be mentioned in the text.

- **This possibility has been expanded on in the results and limitations section of the discussion (lines 390-392).**

Reviewer #2:  Were there any differences in reservoir characteristics by the duration of ART suppression? Also, do we know when ART initiation began and whether earlier ART initiation may have altered subsequent reservoir characteristics?

- **As previously stated, we unfortunately do not have age at ART initiation in the participants, but have data on age at and duration of virologic suppression, for which we have performed additional analyses. We found a positive trend of age at virologic suppression and proviral load across memory and naive CD4+ T cells. Duration of virologic suppression was most correlated to inferred intact HIV-1copies/10^6^ memory CD4+ T cells, suggesting preferential clearance of intact HIV-1 over time. Finally, there was a significant correlation between inferred intact HIV-1 copies/10^6^ naïve CD4+ T cells and CD4 nadir, which may have implications on interaction between immune reconstitution and reservoir expansion in naïve cells. This was added to the results section (lines 206-210, Figure 3).**

- The authors had previously published on the inducibility of the HIV reservoir (JCI Insight) and RNA-Seq + immune phenotyping (Front Immunol) using what seems like the same cohort. Can anything be made of how the size or distribution of the reservoir impact the inducibility of the reservoir and may be influenced by immune exhaustion or other phenotypes?

- **We thank the reviewer for this suggestion. As part of the expanded analyses, we correlated our new IPDA data in memory and naïve CD4+ T cells with the previously published immune activation and exhaustion markers. There was a general trend of positive correlations between intact and total HIV-1 DNA with the proportion of CD25 expressing cells, but inversely correlating with the proportions of CD69 expressing cells. This was noted in the results section (lines 210-215).**

- Is there a way to compare the naïve/memory CD4 IPDA results here with previously published results in those who were infected as adults?

- **We did not perform formal analyses of the IPDA results on the adult published data but this is included in the discussion.**

The integration site analysis seems a bit under-developed. I don’t know that finding 1 proviral integration into ILR2B in one participant represents a particularly solid link. Also, STAT5B and IL2RB were not amongst the highest 10 genes reported with integration sites. Line 222 reference to Supplementary Figure 5 I think is incorrect.

- **Thank you for your suggestions. We have performed additional analyses comparing our integration sites to a previously published integration site dataset in adults. We found that, as in adults, proviral integration is generally enriched in sites with active epigenetic marks (lines 286-292, Supplemental Figure 6). Interestingly, a lower rate of the activating mark, H3K36me3 in activated T cells, was identified in our JH cohort than in adults with non-perinatal infection, although this was not significant after FDR corrections. This was added to the methods and results section with additional references (lines 513-544)**
- **We have also provided our analyses comparing the duration of virologic suppression to the presence of clones, as well as their correlations with the epigenetic marks at the sites of HIV-1 integration. Younger age at virologic suppression and longer duration of virologic suppression was associated with lower percentage of unique integration sites detected once, compared to detection clones (lines 275-279, Supplemental Figure 5).**

Reviewer #3:

The lack of compartmentalization of proviral sequences found in naïve and memory CD4+ T cells is interesting and should be discussed in the context of the existing literature in adults and children. Were common integration sites also detected between subsets of naïve and memory CD4+ T cells?

- **Thank you for the suggestion, we have further explored the data in the context of existing literature in the discussion. Unfortunately, the scarcity of integration sites detected in the naïve CD4+ cells did not allow for comparisons for commonality between the integration sites detected in memory and naïve cells.**

Regarding the “normalization” of viral DNA levels used for Figure 1, it is unclear what dividing viral DNA levels in one cell population by the frequency of this population among the CD4 pool represents. Could you please explain further or replace this transformation by a calculation of the contribution of each subset to the viral DNA in the CD4 pool? Also, if the quantification presented in Fig 1A and B was performed in sorted naïve and memory CD4+ T cells, then the y axis labels should state “copies/10e6 CD4+ T cells” as it does in Fig. 1C and D.

- **This section was clarified to include additional details. Briefly, the proportions of naive CD4+ T cells varied substantially among the participants, with many having high proportions of naïve CD4+ T cell populations (for example, participant 0307 had a proportion of naïve CD4+ T cells of 86% of all CD4+ T cells detected). In previously published research in infant SHIV models (Obregon-Perko et al., J Virol 2020), the effect of this imbalance of cell proportions is that despite lower SHIV DNA copies per million naïve cells compared to SHIV DNA copies per million memory cells, the high percentage of naïve cells results in naïve cells being the largest contributor of proviruses to the latent reservoir. We therefore performed a similar analysis to compare the contributions of the two subsets to the total CD4+ T cell population and found that, despite the high % naïve CD4+ T cells, most proviruses are found in memory cells (lines 180-195).**
- **Because of the calculations in Figure 1A and B vs. C and D, the differences in the Y-axes are intentional. In Figures A and B, the quantities are copies per million naïve or copies per million memory cells. Following normalization of the cell populations in C and D, the quantities are now all copies per million CD4+ T cells, which allows for direct comparisons to the contributions of the subsets within the CD4+ T cell pool. The Y-axis in Figures A and B have been relabeled as Total HIV-1 Copies/10^6^ T cells Subsets and Intact HIV-1 Copies/10^6^ T cell Subsets.**

Additional details regarding the adjustment of the total and intact viral load for memory CD4+ T cell contamination of the sorted naïve cells are needed in the methods section. Was the level of viral DNA in the memory CD4+ T cells taken into consideration for each participant?

- **As discussed above, the potential memory cell contamination has been re-analyzed to include a 95% confidence interval for maximum likelihood of memory cell contamination.**

The gating strategy presented in Fig S2 needs to be corrected. The third plot unlikely represents the CD4+ CD45RA+ CCR7+ population as gated on the first and second plot as a large proportion of CD4- cells remains.

- **The gating strategy has been clarified and corrected in Supplemental Figure 2. The gating strategy used a combination of two different gates: (CD3+ CD4+ CCR7+ CD45RA+), and (CD3+ CD4+ CD95-) as shown in the figure. The arrows have been changed accordingly.**

The authors state both in the introduction and discussion that naïve CD4+ T cells were shown to harbor the majority of DNA in a SHIV macaque model. Similar results were also published using a SIV model.

- **Thank you for the comment, this has been included in the text and references (line 100).**

Line 202-204 the sentence “In addition, […] in eight participants with available DNA” is unnecessary as this is already stated in line 200

- **The extraneous line has been removed for clarity.**

Line 268 of the discussion, the presence of R5-tropic proviruses was also previously reported in children naïve CD4+ T cells

- **This point has been highlighted in the text, and the references have been updated (line 349-350).**

Thank you for allowing us to resubmit our manuscript with the edits and new analyses,

Dr. Deborah Persaud, MD
