# Supplementary material for: Quantification and localization of integrated HIV-1 in memory and naïve CD4+ T cells from adolescents and young adults with perinatally-acquired HIV-1
Source: PLoS Pathog. 2026 Jul 13;22(7):e1014369. doi: 10.1371/journal.ppat.1014369 (PMC13399508; doi:10.1371/journal.ppat.1014369)
Supplement: Peer review history

# Peer review: Decision Letter 0

**DOI:** 10.1371/journal.ppat.1014369.r001

**Authors:** Richard Koup, Mary Kearney

2 Feb 2026

-->PPATHOGENS-D-25-03169

Quantification and localization of integrated HIV-1 in memory and naïve CD4+ T cells from adolescents and young adults with perinatally-acquired HIV-1

PLOS Pathogens

Dear Dr. Persaud,

Thank you for submitting your manuscript to PLOS Pathogens. After careful consideration, we feel that it has merit but does not fully meet PLOS Pathogens's publication criteria as it currently stands. Therefore, we invite you to submit a revised version of the manuscript that addresses the points raised during the review process, including addressing the potential for memory T cell contamination in the naive T cell fractions and how the manuscript builds on previous findings of intact proviruses in naive T cells in children born with HIV.

Please submit your revised manuscript by Apr 03 2026 11:59PM. If you will need more time than this to complete your revisions, please reply to this message or contact the journal office at plospathogens@plos.org. When you're ready to submit your revision, log on to https://www.editorialmanager.com/ppathogens/ and select the 'Submissions Needing Revision' folder to locate your manuscript file.

Please include the following items when submitting your revised manuscript:

* A letter that responds to each point raised by the editor and reviewer(s). You should upload this letter as a separate file labeled 'Response to Reviewers'. This file does not need to include responses to any formatting updates and technical items listed in the 'Journal Requirements' section below.

* A marked-up copy of your manuscript that highlights changes made to the original version. You should upload this as a separate file labeled 'Revised Manuscript with Track Changes'.

* An unmarked version of your revised paper without tracked changes. You should upload this as a separate file labeled 'Manuscript'.

If you would like to make changes to your financial disclosure, competing interests statement, or data availability statement, please make these updates within the submission form at the time of resubmission. Guidelines for resubmitting your figure files are available below the reviewer comments at the end of this letter.

We look forward to receiving your revised manuscript.

Kind regards,

Mary F Kearney

Academic Editor

PLOS Pathogens

Richard Koup

Section Editor

PLOS Pathogens

-->

Sumita Bhaduri-McIntosh

Editor-in-Chief

PLOS Pathogens

orcid.org/0000-0003-2946-9497

Michael Malim

-->Editor-in-Chief

PLOS Pathogens

orcid.org/0000-0002-7699-2064

**Journal Requirements:**

If the reviewer comments include a recommendation to cite specific previously published works, please review and evaluate these publications to determine whether they are relevant and should be cited. There is no requirement to cite these works unless the editor has indicated otherwise.

1) Please ensure that the CRediT author contributions listed for every co-author are completed accurately and in full.

At this stage, the following Authors/Authors require contributions: Adit Dhummakupt, Alexander G Mcfarland, Joseph Szewczyk, John Everett, Carole Lee, Aoife M Roche, Soumia K Bekka, Thuy Anderson, Allison L Agwu, Frederic D Bushman, and Deborah Persaud. Please ensure that the full contributions of each author are acknowledged in the "Add/Edit/Remove Authors" section of our submission form.

The list of CRediT author contributions may be found here: https://journals.plos.org/plospathogens/s/authorship#loc-author-contributions

2) We ask that a manuscript source file is provided at Revision. Please upload your manuscript file as a .doc, .docx, .rtf or .tex. If you are providing a .tex file, please upload it under the item type u2018LaTeX Source Fileu2019 and leave your .pdf version as the item type u2018Manuscriptu2019.

3) Please upload all main figures as separate Figure files in .tif or .eps format. For more information about how to convert and format your figure files please see our guidelines:

https://journals.plos.org/plospathogens/s/figures

4) We have noticed that you have uploaded Supporting Information files, but you have not included a list of legends. Please add a full list of legends for your Supporting Information files after the references list.

5) In the online submission form, you indicated that your data will be submitted to a repository upon acceptance. We strongly recommend all authors deposit their data before acceptance, as the process can be lengthy and hold up publication timelines. Please note that, though access restrictions are acceptable now, your entire minimal dataset will need to be made freely accessible if your manuscript is accepted for publication. This policy applies to all data except where public deposition would breach compliance with the protocol approved by your research ethics board. If you are unable to adhere to our open data policy, please kindly revise your statement to explain your reasoning and we will seek the editor's input on an exemption.

6) Please amend your detailed Financial Disclosure statement. This is published with the article. It must therefore be completed in full sentences and contain the exact wording you wish to be published.

1) State the initials, alongside each funding source, of each author to receive each grant. For example: "This work was supported by the National Institutes of Health (####### to AM; ###### to CJ) and the National Science Foundation (###### to AM)."

2) State what role the funders took in the study. If the funders had no role in your study, please state: "The funders had no role in study design, data collection and analysis, decision to publish, or preparation of the manuscript."

3) If any authors received a salary from any of your funders, please state which authors and which funders..

If you did not receive any funding for this study, please simply state: u201cThe authors received no specific funding for this work.u201d

7)  Please ensure that the funders and grant numbers match between the Financial Disclosure field and the Funding Information tab in your submission form. Note that the funders must be provided in the same order in both places as well.

**Reviewers' Comments:**

Reviewer's Responses to Questions

**Part I - Summary**

Please use this section to discuss strengths/weaknesses of study, novelty/significance, general execution and scholarship.

Reviewer #1: This work characterizes provirus populations in sorted naive and memory CD4+ peripheral blood T cells from 10 people with HIV (PWH) who acquired the virus perinatally yet who had been on long-term suppressive ART at the time of sampling as adolescents or young adults (AYA). Principal findings & conclusions from the study include that: (i) although proviruses were found in both naive and memory cells, they were found in much higher frequencies in the latter subset, (ii) inferred or demonstrated-intact proviruses were identified in both subsets using IPDA or NFL sequencing, respectively, at rates comparable to those reported in prior studies, (iii) HIV env proviral sequences were comingled between memory and naive cell sorts and all were R5 tropic, (iv) integrations sites of proviruses mostly in memory cells did not comingle between subsets, demonstrated evidence of clonal expansion, and were notably sometimes found in genes as also observed in prior studies (i.e., STAT5B, BACH2, IL2RB, and a ZNF gene).

The manuscript is well-organized and for the most part clearly written, the figures are also clear, aesthetically pleasing, and nicely summarize the data, and the conclusions are generally supported by the data, which is not overinterpreted. Overall, however, this work closely resembles a study recently published in JCI (Katusiime, et al., Ref #25) in conduct, findings, and conclusions, and is therefore of modest significance and only incrementally advances our understanding of HIV persistence in ADA on ART who acquired HIV at birth.

Reviewer #2: Understanding the cell and integration site features of HIV proviral latency will be critical to efforts for HIV eradication and control. In this manuscript, the investigators turn their focus on those with perinatally-acquired HIV as these represent an understudied population, especially given the preponderance of naïve CD4 cells during infancy and questions about their contribution to the reservoir into adolescents and young adulthood. The authors studied 10 young adults who were perinatally infected. They performed cell sorting and assessed the reservoir size by total DNA and IPDA levels, performed near-full length proviral sequencing, and integration site analysis. They found the largest intact and total HIV DNA reservoir to be present in memory CD4 cells with significantly lower, but detectable frequencies of infection in naïve CD4 cells. NFL sequencing showed intermixing of sequences from memory and naïve CD4 T cell populations. Integration site analysis was performed in 4 participants and the majority of integration sites were in transcription units and introns. Clonal proliferation was also confirmed.

This study adds to the literature in an understudied area - the distribution and characteristics of the HIV reservoir in HIV perinatally-infected children. The results are of interest to the field and show that while naïve T cells can be infected, the preponderance of infected cells are in the memory CD4 T cell population. This study does notable limitations, including the small numbers of participants and sequences isolated (only an average of 1-2 NFL proviral sequences per participant from naïve T cells and only 1 participant had intact proviruses sequenced from the naïve CD4 population). Integration site analysis does not differentiate between intact and defective proviruses and given the NFL sequencing results, it’s likely that the vast majority of integration sites are for defective proviruses.

Reviewer #3: This study characterizes HIV persistence in naïve and memory CD4+ T cells from adolescents and young adults with ART-suppressed perinatally-acquired HIV at a single time point using Intact Proviral DNA Assay, near-full length sequencing and integration site analysis. As previously reported in infants and adults, in this cohort most of the persisting virus was present in memory cells and showed evidence of clonal proliferation. Interestingly, the authors report the presence of intact proviruses in the naïve population of CD4+ T cells and at a higher proportion than in memory CD4+ T cells.

How the uniqueness of the pediatric immune system influences HIV reservoir establishment remains unknown. The study presented here addresses this critical question with a warranted focus on naïve cells that represent the vast majority of the CD4+ T cell pool during infancy and childhood. Overall, the study seems well-conducted using state-of-the-art assays; the manuscript is well-written and appropriately describes the limitations of the study. While most main results have been previously reported, the number of participants and/or proviruses analyzed is usually limited and additional data are welcome. Furthermore, the cohort selected here is of special interest with a reservoir established perinatally and maintained through 10 to 23 years.

**********

**Part II – Major Issues: Key Experiments Required for Acceptance**

Please use this section to detail the key new experiments or modifications of existing experiments that should be absolutely required to validate study conclusions.

Generally, there should be no more than 3 such required experiments or major modifications for a "Major Revision" recommendation. If more than 3 experiments are necessary to validate the study conclusions, then you are encouraged to recommend "Reject".

Reviewer #1: The significance of the work would be greatly enhanced by uncovering some new insight from existing data or through additional experimentation - probably the latter. Correlative integration site/gene-expression analysis or matching intact proviruses to sites of integration might serve in this respect, though this cannot be predicted with certainly and will no doubt be made more more challenging by the demonstrated difficulty in finding and characterizing naive T cell integration sites in these samples.

Moreover, since proviral persistence in naive CD4+ T cells is one of the principal findings of this work, the possiblity that apparently infected naive cells are actually contaminated memory cells must be more convincingly ruled out by statistical analysis that is more rigorous that that provided in Results. Specifically, the authors should calculate 95% confidence intervals for naive T cell infection frequencies based on sort purities, memory cell infection frequencies, and other factors where measured frequencies greater that this interval cannot reasonably be explained by infected memory cell contamination alone. Details for performing this statistical analysis are provided in Katusiime, et al.

Reviewer #2: - Were there any differences in reservoir characteristics by the duration of ART suppression? Also, do we know when ART initiation began and whether earlier ART initiation may have altered subsequent reservoir characteristics?

- The authors had previously published on the inducibility of the HIV reservoir (JCI Insight) and RNA-Seq + immune phenotyping (Front Immunol) using what seems like the same cohort. Can anything be made of how the size or distribution of the reservoir impact the inducibility of the reservoir and may be influenced by immune exhaustion or other phenotypes?

- Is there a way to compare the naïve/memory CD4 IPDA results here with previously published results in those who were infected as adults?

- The integration site analysis seems a bit under-developed. I don’t know that finding 1 proviral integration into ILR2B in one participant represents a particularly solid link. Also, STAT5B and IL2RB were not amongst the highest 10 genes reported with integration sites. Line 222 reference to Supplementary Figure 5 I think is incorrect.

Reviewer #3: (No Response)

**********

**Part III – Minor Issues: Editorial and Data Presentation Modifications**

Please use this section for editorial suggestions as well as relatively minor modifications of existing data that would enhance clarity.

Reviewer #1: Because ART suppresses ongoing HIV replication, samples taken even after years on ART to some extent reflect the state of the infection immediately prior to ART administration, in this case in an immune environment in which the fraction of CD4+ T cells exceed 90% at birth but declines rapidly with age. Therefore, participant ages at the time of initial ART administration and at the first test of undetectable viremia should be added to Table 1. This would, for example, enable the authors to potentially establish a more granular correlation between participant age at initial viremic suppression and naive T cell contribution to the pool of infected T cells at the time of sampling, which would strengthen the work considerably.

Please refer to IPDA+ proviruses as "potentially intact", "inferred intact", or similar, rather than "intact". "Intact" or "genetically intact" should only refer to proviruses that have been sequenced and verified as capable of producing new virus by some established standard. Intact proviral sequences should also be submitted to GenBank.

Phylogenetic co-mingling of env sequences from naive and memory cells sorts may be a result of memory cell contamination. This possibility should be mentioned in the text.

Reviewer #2: (No Response)

Reviewer #3: • The lack of compartmentalization of proviral sequences found in naïve and memory CD4+ T cells is interesting and should be discussed in the context of the existing literature in adults and children. Were common integration sites also detected between subsets of naïve and memory CD4+ T cells?

• Regarding the “normalization” of viral DNA levels used for Figure 1, it is unclear what dividing viral DNA levels in one cell population by the frequency of this population among the CD4 pool represents. Could you please explain further or replace this transformation by a calculation of the contribution of each subset to the viral DNA in the CD4 pool? Also, if the quantification presented in Fig 1A and B was performed in sorted naïve and memory CD4+ T cells, then the y axis labels should state “copies/10e6 CD4+ T cells” as it does in Fig. 1C and D.

• Additional details regarding the adjustment of the total and intact viral load for memory CD4+ T cell contamination of the sorted naïve cells are needed in the methods section. Was the level of viral DNA in the memory CD4+ T cells taken into consideration for each participant?

• The gating strategy presented in Fig S2 needs to be corrected. The third plot unlikely represents the CD4+ CD45RA+ CCR7+ population as gated on the first and second plot as a large proportion of CD4- cells remains.

• The authors state both in the introduction and discussion that naïve CD4+ T cells were shown to harbor the majority of DNA in a SHIV macaque model. Similar results were also published using a SIV model.

• Line 202-204 the sentence “In addition, […] in eight participants with available DNA” is unnecessary as this is already stated in line 200

• Line 268 of the discussion, the presence of R5-tropic proviruses was also previously reported in children naïve CD4+ T cells

**********

PLOS authors have the option to publish the peer review history of their article (what does this mean?). If published, this will include your full peer review and any attached files.

If you choose “no”, your identity will remain anonymous but your review may still be made public.

**Do you want your identity to be public for this peer review?**  For information about this choice, including consent withdrawal, please see our Privacy Policy.

Reviewer #1: No

Reviewer #2: No

Reviewer #3: No

**Figure resubmission:**

-->While revising your submission, we strongly recommend that you use PLOS’s NAAS tool (https://ngplosjournals.pagemajik.ai/artanalysis) to test your figure files. NAAS can convert your figure files to the TIFF file type and meet basic requirements (such as print size, resolution), or provide you with a report on issues that do not meet our requirements and that NAAS cannot fix.-->

After uploading your figures to PLOS’s NAAS tool - https://ngplosjournals.pagemajik.ai/artanalysis, NAAS will process the files provided and display the results in the "Uploaded Files" section of the page as the processing is complete. If the uploaded figures meet our requirements (or NAAS is able to fix the files to meet our requirements), the figure will be marked as "fixed" above. If NAAS is unable to fix the files, a red "failed" label will appear above. When NAAS has confirmed that the figure files meet our requirements, please download the file via the download option, and include these NAAS processed figure files when submitting your revised manuscript.

**Reproducibility:**

To enhance the reproducibility of your results, we recommend that authors of applicable studies deposit laboratory protocols in protocols.io, where a protocol can be assigned its own identifier (DOI) such that it can be cited independently in the future. Additionally, PLOS ONE offers an option to publish peer-reviewed clinical study protocols. Read more information on sharing protocols at https://plos.org/protocols?utm_medium=editorial-email&utm_source=authorletters&utm_campaign=protocols-->

# Author comment: Author response to Decision Letter 1

**DOI:** 10.1371/journal.ppat.1014369.r002

6 May 2026

Attachment Submitted filename: Reviewer comment responses 20260504.docx

# Peer review: Decision Letter 1

**DOI:** 10.1371/journal.ppat.1014369.r003

**Authors:** Richard Koup, Mary Kearney, Richard Koup, Mary Kearney

11 Jun 2026

Dear Dr. Persaud,

We are pleased to inform you that your manuscript 'Quantification and localization of integrated HIV-1 in memory and naïve CD4+ T cells from adolescents and young adults with perinatally-acquired HIV-1' has been provisionally accepted for publication in PLOS Pathogens.

Before your manuscript can be formally accepted you will need to complete some formatting changes, which you will receive in a follow up email. A member of our team will be in touch with a set of requests.

Please note that your manuscript will not be scheduled for publication until you have made the required changes, so a swift response is appreciated.

IMPORTANT: The editorial review process is now complete. PLOS will only permit corrections to spelling, formatting or significant scientific errors from this point onwards. Requests for major changes, or any which affect the scientific understanding of your work, will cause delays to the publication date of your manuscript.

Should you, your institution's press office or the journal office choose to press release your paper, you will automatically be opted out of early publication. We ask that you notify us now if you or your institution is planning to press release the article. All press must be co-ordinated with PLOS.

Thank you again for supporting Open Access publishing; we are looking forward to publishing your work in PLOS Pathogens.

Best regards,

Mary F Kearney

Academic Editor

PLOS Pathogens

Richard Koup

Section Editor

PLOS Pathogens

Sumita Bhaduri-McIntosh

Editor-in-Chief

PLOS Pathogens

orcid.org/0000-0003-2946-9497

Michael Malim

Editor-in-Chief

PLOS Pathogens

orcid.org/0000-0002-7699-2064

***********************************************************

Reviewer Comments (if any, and for reference):

Reviewer's Responses to Questions

**Part I - Summary**

Please use this section to discuss strengths/weaknesses of study, novelty/significance, general execution and scholarship.

Reviewer #1: The authors have addressed concerns raised in the original review and revised the submission to this reviewer's satisfaction.

Reviewer #2: The authors have addressed my concerns.

**********

**Part II – Major Issues: Key Experiments Required for Acceptance**

Please use this section to detail the key new experiments or modifications of existing experiments that should be absolutely required to validate study conclusions.

Generally, there should be no more than 3 such required experiments or major modifications for a "Major Revision" recommendation. If more than 3 experiments are necessary to validate the study conclusions, then you are encouraged to recommend "Reject".

Reviewer #1: (No Response)

Reviewer #2: (No Response)

**********

**Part III – Minor Issues: Editorial and Data Presentation Modifications**

Please use this section for editorial suggestions as well as relatively minor modifications of existing data that would enhance clarity.

Reviewer #1: (No Response)

Reviewer #2: (No Response)

**********

PLOS authors have the option to publish the peer review history of their article (what does this mean?). If published, this will include your full peer review and any attached files.

If you choose “no”, your identity will remain anonymous but your review may still be made public.

**Do you want your identity to be public for this peer review?**  For information about this choice, including consent withdrawal, please see our Privacy Policy.

Reviewer #1: No

Reviewer #2: No

# Editor report: Acceptance letter

**DOI:** 10.1371/journal.ppat.1014369.r004

**Authors:** Richard Koup, Mary Kearney, Richard Koup, Mary Kearney

Dear Dr. Persaud,

We are delighted to inform you that your manuscript, "Quantification and localization of integrated HIV-1 in memory and naïve CD4+ T cells from adolescents and young adults with perinatally-acquired HIV-1," has been formally accepted for publication in PLOS Pathogens.

We have now passed your article onto the PLOS Production Department who will complete the rest of the pre-publication process. All authors will receive a confirmation email upon publication.

The corresponding author will soon be receiving a typeset proof for review, to ensure errors have not been introduced during production. Please review the PDF proof of your manuscript carefully, as this is the last chance to correct any scientific or type-setting errors. Please note that major changes, or those which affect the scientific understanding of the work, will likely cause delays to the publication date of your manuscript. Note: Proofs for Front Matter articles (Pearls, Reviews, Opinions, etc...) are generated on a different schedule and may not be made available as quickly.

Soon after your final files are uploaded, the early version of your manuscript, if you opted to have an early version of your article, will be published online. The date of the early version will be your article's publication date. The final article will be published to the same URL, and all versions of the paper will be accessible to readers.

For Research Articles, you will receive an invoice from PLOS for your publication fee after your manuscript has reached the completed accept phase. If you receive an email requesting payment before acceptance or for any other service, this may be a phishing scheme. Learn how to identify phishing emails and protect your accounts at https://explore.plos.org/phishing.

Thank you again for supporting open-access publishing; we are looking forward to publishing your work in PLOS Pathogens.

Best regards,

Sumita Bhaduri-McIntosh

Editor-in-Chief

PLOS Pathogens

orcid.org/0000-0003-2946-9497

Michael Malim

Editor-in-Chief

PLOS Pathogens

orcid.org/0000-0002-7699-2064
